# Genomic insight into the origin, domestication, dispersal, diversification and human selection of Tartary buckwheat

**DOI:** 10.1186/s13059-024-03203-z

**Published:** 2024-02-27

**Authors:** Yuqi He, Kaixuan Zhang, Yaliang Shi, Hao Lin, Xu Huang, Xiang Lu, Zhirong Wang, Wei Li, Xibo Feng, Taoxiong Shi, Qingfu Chen, Junzhen Wang, Yu Tang, Mark A. Chapman, Mateja Germ, Zlata Luthar, Ivan Kreft, Dagmar Janovská, Vladimir Meglič, Sun-Hee Woo, Muriel Quinet, Alisdair R. Fernie, Xu Liu, Meiliang Zhou

**Affiliations:** 1 State Key Laboratory of Crop Gene Resources and Breeding, Institute of Crop Sciences, Chinese Academy of Agricultural Sciences, Beijing, 100081 China; 2 Tibet Key Experiments of Crop Cultivation and Farming/College of Plant Science, Tibet Agriculture and Animal Husbandry University, Linzhi, 860000 China; 3 Research Center of Buckwheat Industry Technology, Guizhou Normal University, Guiyang, 550001 China; 4 Xichang Institute of Agricultural Science, Liangshan Yi People Autonomous Prefecture, Liangshan, Sichuan, 615000 China; 5 Biological Sciences, University of Southampton, Life Sciences Building 85, Highfield Campus, Southampton, SO17 1BJ UK; 6 Biotechnical Faculty, University of Ljubljana, Jamnikarjeva 101, SI-1000 Ljubljana, Slovenia; 7 Nutrition Institute, Koprska Ulica 98, SI-1000 Ljubljana, Slovenia; 8 Gene Bank, Crop Research Institute, Drnovská 507, Prague 6, Czech Republic; 9 Agricultural Institute of Slovenia, Hacquetova ulica 17, SI-1000 Ljubljana, Slovenia; 10 Department of Crop Science, Chungbuk National University, Cheong-ju, Republic of Korea; 11 Groupe de Recherche en Physiologie Végétale (GRPV), Earth and Life Institute-Agronomy (ELI-A), Université catholique de Louvain, Croix du Sud 45, boîte L7.07.13, B-1348 Louvain-la-Neuve, Belgium; 12 Department of Molecular Physiology, Max-Planck-Institute of Molecular Plant Physiology, 14476 Potsdam, Germany

**Keywords:** Domestication, Migration, Artificial selection, Buckwheat, Genomics

## Abstract

**Background:**

Tartary buckwheat, *Fagopyrum tataricum*, is a pseudocereal crop with worldwide distribution and high nutritional value. However, the origin and domestication history of this crop remain to be elucidated.

**Results:**

Here, by analyzing the population genomics of 567 accessions collected worldwide and reviewing historical documents, we find that Tartary buckwheat originated in the Himalayan region and then spread southwest possibly along with the migration of the Yi people, a minority in Southwestern China that has a long history of planting Tartary buckwheat. Along with the expansion of the Mongol Empire, Tartary buckwheat dispersed to Europe and ultimately to the rest of the world. The different natural growth environments resulted in adaptation, especially significant differences in salt tolerance between northern and southern Chinese Tartary buckwheat populations. By scanning for selective sweeps and using a genome-wide association study, we identify genes responsible for Tartary buckwheat domestication and differentiation, which we then experimentally validate. Comparative genomics and QTL analysis further shed light on the genetic foundation of the easily dehulled trait in a particular variety that was artificially selected by the Wa people, a minority group in Southwestern China known for cultivating Tartary buckwheat specifically for steaming as a staple food to prevent lysine deficiency.

**Conclusions:**

This study provides both comprehensive insights into the origin and domestication of, and a foundation for molecular breeding for, Tartary buckwheat.

**Supplementary Information:**

The online version contains supplementary material available at 10.1186/s13059-024-03203-z.

## Background

The current appearance of crops is the result of the combined action of their natural and cultural environments [1]. During long-term crop domestication, allelic variations with desired qualities in traits such as yield, taste, and cultivation practices were artificial selected [2]. When these domesticated crops spread to broader geographical areas through human migration, only those types adapted to their new environment and of use to humans would be selected, leading to the gradual expansion of the proportion of the allelic variations within the population, and ultimately differentiation into diverse germplasm resources [3, 4]. The diverse germplasm resources also lead to different dietary habits, creating unique cultural environments for human concentrated communities in different regions [1]. Thus, the study of the genetic basis of crop domestication not only helps to promote crop genetic improvement, but also contributes to a comprehensively understanding of the history and development of modern agricultural societies.

Buckwheat belongs to the Polygonaceae family, which is known for its abundant pharmaceutical plants, including *Polygonum multiflorum* and *Rheum officinale*. These pharmaceutical plants are rich in various bioactive substances with health promoting effects. As the food crop with the closest phylogenetic relationship to these pharmaceutical plants, buckwheat is generally considered to have more abundant bioactive substances than other more widespread main grain crops of the Poaceae [5]. Besides these health promoting effects, these substances are usually present due to their role in plant defense against biotic and abiotic stress [6, 7]. At present, there are two most widely cultivated buckwheat species, including self-pollinated Tartary buckwheat and self-incompatible common buckwheat [8]. The self-pollinated nature of Tartary buckwheat makes it more suitable for genetic diversity research than common buckwheat. Meanwhile, it is generally considered that Tartary buckwheat exhibited greater health protection efficacy and high-altitude adaptability than common buckwheat [9]. According to pharmaceutical classics such as 'Compendium of Materia Medica', 'Qian Jin Yao Fang', and 'Dictionary of Traditional Chinese Medicine', Tartary buckwheat has health beneficial effects such as calming the mind, strengthening the heart, anti-inflammatory bioactivities as well as the ability to promote weight loss. However, compared to wild accessions, domesticated Tartary buckwheat bear as a common set of traits, known as the domestication syndrome, which includes loss of seed shattering, increased seed size and reduced seed dormancy [10]. Along with changes in these visible traits, a lower level of many bioactive compounds has been selected for, likely due to the fact that they are usually bitter in taste [11, 12]. Given this, study of the domestication history of Tartary buckwheat will improve the understanding of the genetic basis of the accumulation of bioactives as well as the utilization of wild buckwheat for molecular breeding.

The unique natural characteristics of Tartary buckwheat and not being a member of the Poaceae distinguish it from the major grain crops, increasing the interest in its domestication history. De Candolle initially speculated that it originated in northern China. However, no one has confirmed the distribution of wild buckwheat in the region, leading to this speculation is not widely accepted [13]. Subsequently, using molecular markers, Ohnishi speculated that Tartary buckwheat originated in the eastern part of Tibet and the neighboring areas of Yunnan and Sichuan [14, 15]. Although the historiography, morphology, reproductive biology and the distribution of wild relatives supports this hypothesis [16, 17], more molecular evidence is still needed to confirm this hypothesis, as these studies were only based on limited allozyme variability and amplified fragment length polymorphism (AFLP) obtained from a small number of Tartary buckwheat accessions. In addition, there is still great controversy regarding the domestication and dispersal history of buckwheat. Linguistic evidence suggests that the Chinese name of buckwheat was borrowed from eastern Tibeto-Burman speakers to the south-west of the Han Chinese [18], suggesting a close relationship between southwestern China and buckwheat. Moreover, the English name Tartary is derived from Tatars, which is the name of Mongols according to 'Marco Polo's Travels', 'Dell'Historia della China', and 'Matteo Ricci's Reading Notes about China', also indicate a close relationship between European buckwheat and Mongolia. Morphology and geographical distribution additionally suggest that buckwheat cultivation began in southwestern China [19, 20]. However, palynological and archaeological records suggest that buckwheat cultivation may started in northern China [21], and was probably introduced into central and western Europe through Siberia 1,500 years ago [21, 22]. Given the difficulty of finding ancient buckwheat seeds and the inability to distinguish the pollen fossils of wild and cultivated buckwheat [23], the origin, domestication, and dispersal history of buckwheat remain to be resolved.

The development of genomics has promoted a comprehensive understanding of the origin of crop domestication, filling in the gaps left by traditional archaeology [24, 25]. At present, there has been systematic research on the origin of grain crops such as rice [26–28], maize [29, 30], vegetables such as *Brassica juncea* [31], lettuce [32], and fruits such as grapevine [33] as well as protein-rich legumes such as common bean [34] and chickpea [35]. Previously, based on the phylogenetic map and genetic differentiation of Tartary buckwheat germplasm resources, we found Tartary buckwheat might have migrated from northern China to other countries [8]. However, due to the difficulty in obtaining wild and outside China Tartary buckwheat germplasm resources, the origin and domestication history of Tartary buckwheat remains unclear. In the present study, by supplementing accessions collected in potential places of origin with material from other areas, the origin and domestication history of Tartary buckwheat was revealed. By scanning selective sweeps and genome-wide association studies for disease resistance and salt stress resistance, genes implicated in domestication and adaptability diversification were illuminated. Comparative genomics and QTL analysis further elucidated the genetic basis of domestication of the only Tartary buckwheat variety harboring a readily dehulled phenotype. These results provide a valuable resource for Tartary buckwheat molecular breeding and the understanding of the history of agriculture and aspects of civilization linked thereto.

## Results

### A Himalayan origin of Tartary buckwheat

**Fig. 1:**
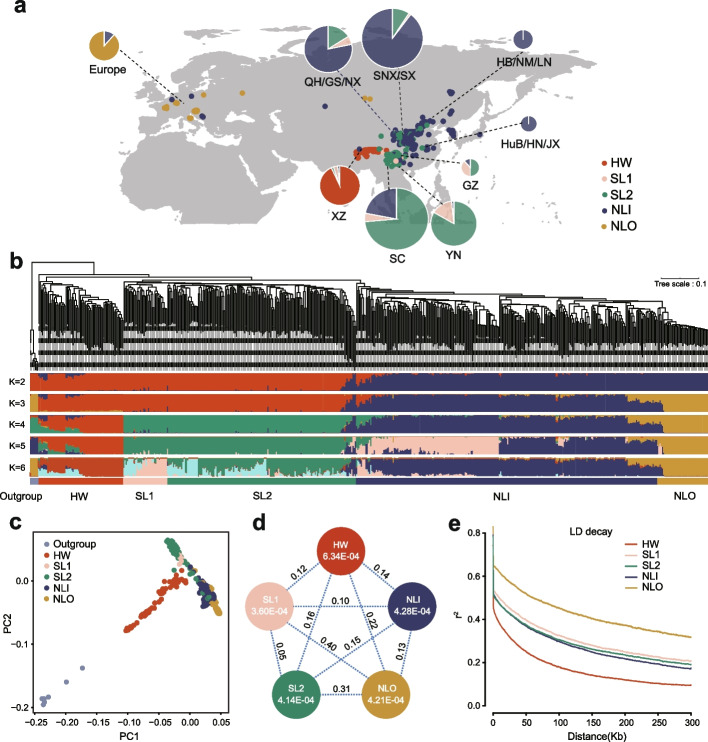
Geographic distribution, population structure and genomic diversity of Tartary buckwheat accessions. Geographic distributions of 567 Tartary buckwheat accessions. The radius of each pie represents the sample size in each region and the colors indicate the proportions of HW (Himalayan wild accession), SL1 (Southwest landrace 1), SL2 (Southwest landrace 2), NLI (Northern Landrace-Within China), NLO (Northern Landrace-Outside China). XZ, Xizang Province; SC, Sichuan Province; YN, Yunnan Province; GZ, Guizhou Province; HuB/HN/JX, Hubei/Hunan/Jiangxi Province; HB/NM/LN, Hebei/Inner Mongolia/Liaoning Province; SNX/SX, Shannxi/Shanxi Province; QH/GS/NX, Qianghai/Gansu/Ningxia Province. **B** The maximum-likelihood phylogeny of 567 Tartary buckwheat accessions and model-based clustering analysis with different numbers of ancestry kinship (K= 2-6). Different colors indicate different groups based on the population structure. **C** PCA plots of 567 Tartary buckwheat accessions and outgroup. Colors represent the membership at K = 6 (Fig. 1b). **D** Nucleotide diversity (*π*; within circles) and population divergence (*F*_ST_; between circles) for the five groups (the outgroup population was not included). **E** Group-specific LD decay plots

To explore Tartary buckwheat center of origin, we have collected genome-wide resequencing data for 567 accessions collected from 17 countries representing various geographical regions (Fig. 1a; Additional file 1: Tables S1, S2). Among them, 78 accessions were newly described in this study, which included 41 wild accessions from the Himalayan region, 36 landraces collected from areas outside the current border of China, and one representative landrace with an easily-dehulled-phenotype collected from southwest China. By contrast 496 accessions were described in a previous study [8]. We then performed phylogenetic and genetic structure analyses of the Tartary buckwheat population, examining two to six clusters (K) (Fig. 1b). At K = 6, the outgroup forms its own group, and Tartary buckwheat was optimally characterized by the presence of five major clusters. Three clusters are similar to those found previously [8], i.e., accessions collected from the Himalayan region formed Himalayan wild (HW) group, accessions mainly collected from southwestern China formed Southwestern landraces (SL) group, accessions mainly collected from northern China formed Northern landraces (NL) group. In addition, NL landraces splitted into two groups in our analysis (one group of NL within China landraces [NLI] and the newly sequenced NL outside China landraces [NLO]), and the SL group divided into two sub-groups, namely SL1 and SL2. The newly added wild accessions grouped with the HW group. The clustering based on K = 2 illustrated the previously reported strong north-south divide. NLI group divided into two subgroups (K = 5) while merged as one (K = 6). The principal component analysis (PCA) revealed a similar population structure compared to the evolutionary tree analysis (Fig. 1c). The population structure shown here is consistent with that in previous research [8].

Nucleotide diversity (*π*) and population fixation statistics (*F*_ST_) were subsequently estimated in five major groups (Fig. 1d; Additional file 1: Table S3; Additional file 2: Fig. S1, S2). The HW group (Himalayan accessions mainly grouped) exhibited higher genetic diversity compared to SL group (Yunnan and Sichuan province accessions mainly grouped) and NL group (northern China accessions mainly grouped). The *F*_ST_ between NLO and NLI is smaller than that between NLO and other groups, supporting the hypothesis that Tartary buckwheat was spread from northern China to Europe. Linkage disequilibrium (LD) decayed faster in the HW group than other groups (Fig. 1e), which was consistent with the highest *π* in HW, confirming that the Himalayan region is more likely to be the origin center of cultivated Tartary buckwheat compared to northern China and Sichuan or Yunnan province in southern China. The LD in the NLO subgroup decayed slower than that in NLI, which might be expected given that the NLO accessions have been selectively bred and improved, which is consistent with the genetic diversity and population fixation statistics. In summary, these results demonstrate that Tartary buckwheat originated in the Himalayan region, and subsequently domesticated, forming the SL and NL groups, respectively.

### Dispersal of Tartary buckwheat followed routes of human migration

**Fig. 2:**
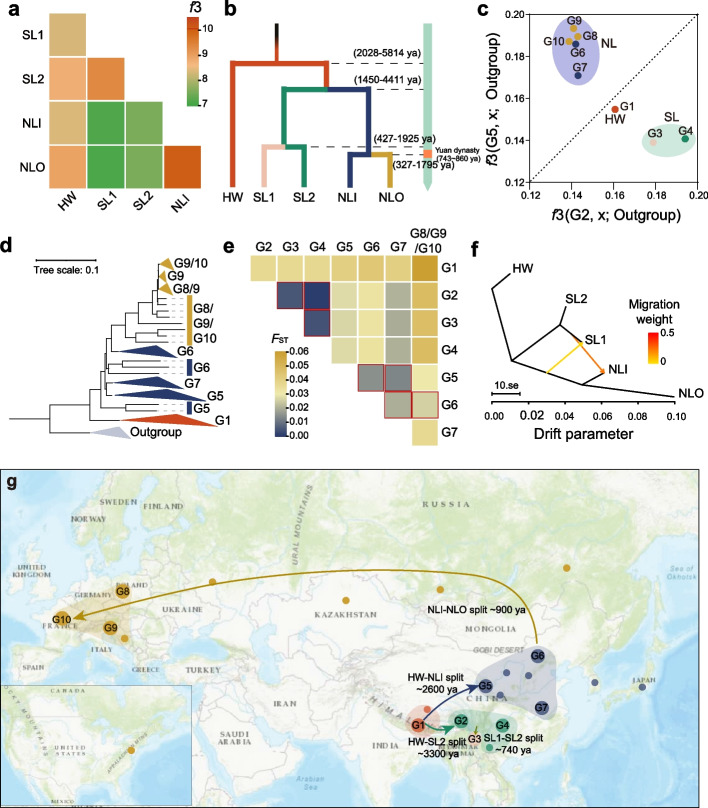
Demographic history and dispersal of Tartary buckwheat. **A** Heatmap showing the similarity of five population through outgroup *f*_3_ matrix. **B** Divergence times of the five populations. The range of predicted divergence time was shown. **C** Outgroup *f*_3_ statistics biplot measuring genetic similarity. Diagonal line marks the *f*_3_ statistics for G2/G5. Different groups representing accessions collected from different areas. G1, Himalayan region; G2, Sichuan; G3, Yunnan; G4, Guizhou; G5, Qinghai-Gansu; G6, Inner Mongolia-Hebei; G7, Hunan-Hubei-Jiangxi; G8, Poland; G9, Slovenia and G10, France. **D** Phylogenic tree of outgroup, Himalayan located group (G1), northern China located group (G5-G7) and outside China located group (G8-G10). **E** Pairwise fixation index (*F*_ST_) of the mini-groups of Tartary buckwheat. **F** Gene flow between populations estimated using Treemix. Yellow and orange lines between populations indicate gene flow. **G** The possible spread of Tartary buckwheat from its origins in the Himalayas. Ten groups representing the population along the route are indicated. The average predicted divergent times are shown

Human migration has promoted the spread of many cultivated crops [1]. Population structure analysis suggested a Himalayan origin and divergent selection of Tartary buckwheat (Fig. 1). To further investigate the possible dispersal history of Tartary buckwheat, the population relationship was further analyzed using *f*_3_ statistics, with other *Fagopyrum* species as the outgroup. The results further confirm the close relationship between SL1 and SL2 and between NLI and NLO and the relatively distant relationship between NL and SL groups (Fig. 2a), in accordance with the population structure (Fig. 1). Then, using qpGraph analysis to consider the potential population mixing events (Additional file 2: Fig. S3), similar relationships between subgroups in SL and NL were found, suggesting the reliability of the grouping.

Subsequently, SMC++ was used to estimate the divergence time (Fig. 2b; Additional file 2: Fig. S4) among the five populations. Cultivated accessions diverged from the HW group around 2,028-5,814 years ago, which coincides with the time when the Yi people migrated from Tibet to the Sichuan province [23]. According to the Yi classic 'Southwest Yi Annals', the ancestors of the Yi people migrated from the Himalayan region, seemingly bringing Tartary buckwheat to Sichuan province. Subsequently, the SL and NL groups differentiated approximately 1,450-4,411 years ago. The SL1/SL2 populations and the NLI/NLO groups diverged at a similar time, ca. 300-1,900 YBP, which was in accordance with the time of the westward expansion of the Mongol Empire. The result of effective population size (*Ne*; Additional file 2: Fig. S5) exhibited similar divergent time. We therefore speculate that Tartary buckwheat spread to Europe with the expansion of the Mongol Empire, which was also illustrated in 'The History of The Mongol Empire'.

To evaluate the accuracy of the candidate dispersal route of Tartary buckwheat, we divided Tartary buckwheat accessions into ten mini-groups based on geographical distribution. The silhouette score based on genotype showed the groups can be well clustered (Additional file 2: Fig. S6). The *f*_3_ statistics revealed the genetic relationship between HW and SL is comparable to that between HW and NL, suggesting HW is the common ancestor of SL and NL groups (Fig. 2c). The accessions collected from outside China (G8-G10) have closer genetic relationship with NL group (G5-G7) compared to HW (G1) and SL groups (G2-G4). The phylogenetic tree showed that compared to individuals distributed in northern China (G5-G7) and outside China (G8-G10), individuals in G1 (located in Himalayan region) possess a closer genetic relationship with outgroup (Fig. 2d). And individuals in G5 (located in Qinghai-Gansu) were closer to their ancestors than other individuals in NL group, which was in accordance with the dispersal route of Tartary buckwheat from the Himalayas to northern China. Not only phylogenetic tree (Fig. 2d) but also pairwise fixation index (Fig. 2e) showed that individuals in NLO (G8-G10) have closer relationships with G6 (Inner Mongolia-Hebei) than other mini-groups in NL (G5 and G7, located in Qinghai, Gansu, Hunan, Hubei and Jiangxi province), supporting the hypothesis that Tartary buckwheat spread to Europe through the Mongolian region.

In cases where populations are not geographically isolated admixture and introgression can occur, and in some cases this can be adaptive [36]. TreeMix identified two instances of gene flow among the five subpopulations, namely a substantial migration from SL1 to NLI and a lesser migration from the NLI/NLO ancestor to SL1 (Fig. 2f; Additional file 2: Fig. S7). The *f*_*dM*_ analysis additionally reveals that the SL1 population introgressed more genetic components into NLI than NLO (Additional file 1: Table S4; Additional file 2: Fig. S8). D-statistics found that NLI accessions located in Hunan-Hubei-Jiangxi province (G7) were characterized by substantial introgressions from accessions located in Qinghai (G5; |Z score| = 4.09, *P* = 4.26×10^-5^) and Inner Mongolia province (G6; |Z score| = 10.2, *P* = 2.24×10^-24^), possibly due to the close geographical proximity (Additional file 1: Table S5). Such large-scale gene transfer may enhance the genetic diversity of the accessions.

Subsequently, a pattern diagram displaying the dispersal route of Tartary buckwheat was summarized (Fig. 2g). About 3,300 years ago, possibly with the migration of the Yi people, Tartary buckwheat spread from the Himalayas to southwestern China. Around 3,000 years ago, Tartary buckwheat spread to northern China. Around 1,500 years ago, the SL1 and SL2 populations differentiated and formed SL1 subgroup with higher domestication degree. Subsequently, possibly with the westward expansion of the Mongol Empire about 1,000 years ago, Tartary buckwheat dispersed from northern China to Europe, ultimately resulting in its current global distribution pattern.

### Selection targets during domestication

**Fig. 3:**
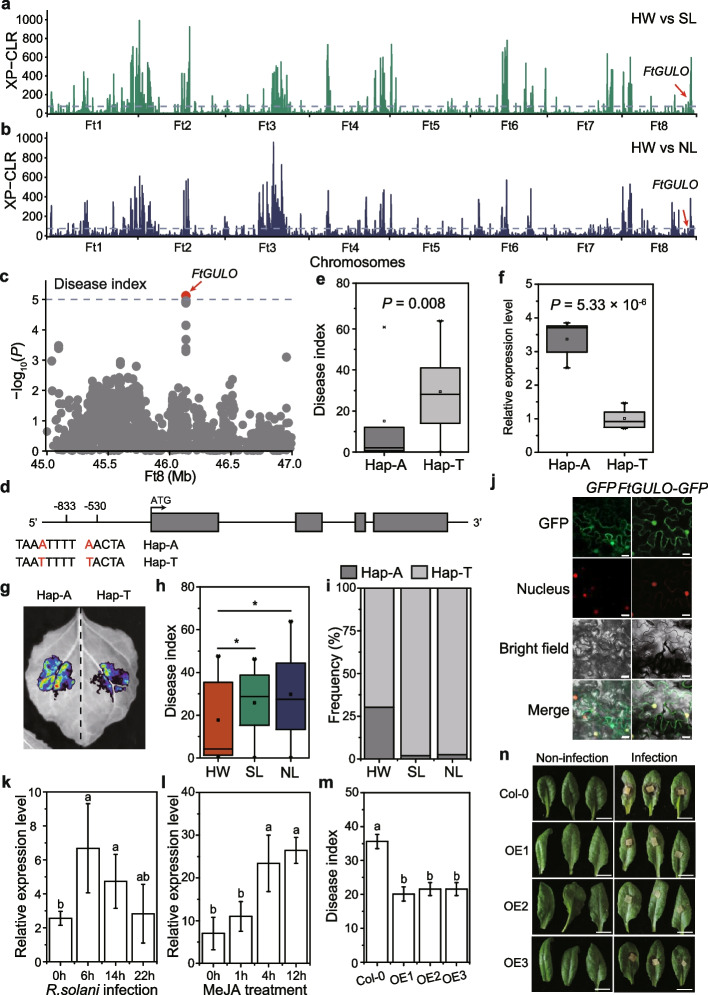
Variation of *FtGULO* controls disease resistance during Tartary buckwheat domestication. **A**-**B** Selective sweeps identified through comparisons between HW and SL (**A**) and HW and NL (**B**) using XP-CLR (cross-population composite likelihood-ratio test). The dashed line represents the top 5% of values therefore scores in these regions were regarded as selective sweeps. **C** Local Manhattan plot of GWAS signals on Chr 8 for resistance to *R. solani* AG4-HGI 3. The dashed line represents the threshold (-log_10_*P* = 5). **D** Schematic diagram of *FtGULO* gene structure. Two SNPs in the promoter of *FtGULO* were marked as red letters and result in haplotypes (Hap) A and T. **E** Box plots show disease index in plants carrying the two haplotypes (Hap). *n*_Hap-A_ = 8, *n*_Hap-T_ = 234. *P* values were calculated using a two-tailed *t*-tests. **F** Expression of *FtGULO* in accessions harboring the two haplotypes. Error bars indicate the ± s.d., *n* = 6. Significance was tested using one-way ANOVA. **G** Transcription activity of *FtGULO* promoters with two haplotypes. **H** Disease index of accessions among HW, NL and SL groups. *n*_HW_ = 10, *n*_NL_ = 96, *n*_SL_ = 140. Significant was tested using two-tailed *t*-tests. *, *P* < 0.05. **I** Frequencies of the two haplotypes in the HW, NL and SL groups. **J** Subcellular localization of *FtGULO*-GFP fusion protein transient expression in *N. benthamiana* leave cells. Scale bars, 10 µm. (K-L) Relative expression levels of *FtGULO* during *R. solani* infection (**K**) and MeJA treatment (**L**). Histone H3 was used as the internal reference. **M** Disease index of *Arabidopsis* lines heterologously expressing *FtGULO*. Significant differences were identified using one-way ANOVA. *n* = 6. **N** Phenotypes of *Arabidopsis* WT lines and lines heterologously expressing *FtGULO* with and without infection with *R. solani* AG4-HGI 3. Scale bars, 1 cm

To identify potential selective signals involved in the domestication of Tartary buckwheat, we performed the cross-population composite likelihood ratio test (XP-CLR) between HW and SL (Fig. 3a) and between HW and NL (Fig. 3b). We identified genomic regions in the top 5% of the distribution of XP-CLR values which revealed 404 sweeps containing 2,909 genes in the HW-SL comparison and 415 sweeps containing 2,793 genes in HW-NL (Additional file 1: Table S6, S7). Among them, 1,282 genes overlapped in both comparisons (Additional file 1: Table S8; Additional file 2: Fig. S9). The remaining 1,627 (56% of the candidate genes) in HW-SL and 1,511 (54%) in HW-NL represent those with divergent histories since the origin of domesticated Tartary buckwheat. Only 330 genes located in 44 selective sweeps in HW-SL comparison and 317 genes located in 78 selective sweeps in HW-NL comparison were overlapped with previous study. This was because more than half of HW accessions and 10% of the NL accessions were newly added in this study. In addition, de-correlated composite of multiple signals (DCMS) approach was also used to identify selective sweeps. 2,803 genes in 410 selective sweeps were identified in HW-SL comparison, and 3,377 genes in 487 selective sweeps were identified HW-NL comparison (Additional file 1: Table S9, S10). Only 785 genes were overlapped in both comparisons (Additional file 1: Table S11), further confirming the independent domestication process.

Many genes selected during domestication in both SL and NL are potentially involved in domestication related traits (Additional file 1: Table S8). For instance, a receptor-like protein kinase [37] was a key gene regulating plant height. Thioredoxin [38], pathogenesis-related protein [39] and remorin [40] were well-known plant disease resistance associated genes while some homologous of GRAS transcription factors [41] has previously been defined as being involved in grain weight regulation. The identification of these domestication trait related genes provides a genetic basis for the mechanism underlying Tartary buckwheat domestication.

*Rhizoctonia solani* AG4-HGI 3 is a devastating soil-borne pathogen that seriously threatens Tartary buckwheat cultivation [7]. Previous research demonstrated the content of metabolites associated with disease resistance decreased during Tartary buckwheat domestication [12]. We therefore investigated whether genes responsible for resistance to *R. solani* underwent selection during Tartary buckwheat domestication. Notably, one significant locus identified by GWAS of disease resistance [7] was found to have undergone selection during domestication of the NL and SL groups (Fig. 3c; Additional file 1: Table S12; Additional file 2: Fig. S10). Haplotype analysis identified two variants located at 833 bp and 530 bp in the promoter of a gene encoding L-gulonolactone oxidase (*FtGULO*, *FtPinG0809053200*), which is involved in ascorbate biosynthesis (Fig. 3d) [42, 43]. Phylogenetic analysis demonstrates this gene is an orthologue of L-gulonolactone oxidase in other species (Additional file 2: Fig. S11). Accessions harboring the A-haplotype exhibited higher disease resistance and higher *FtGULO* expression compared to those harboring the T-haplotype (Fig. 3e, f), suggesting *FtGULO* is an important locus underlying resistance to *R. solani* AG4-HGI 3 in Tartary buckwheat. Transient activation assays demonstrate that higher LUC expression in leaves transient expressing promoters of the A-haplotype compared to those of the T-haplotype, confirming the natural variations in the promoter of *FtGULO* were involved in Tartary buckwheat disease resistance (Fig. 3g). The disease index was significantly greater in the SL and NL groups compared to HW (Fig. 3h), confirming disease resistance decreased during Tartary buckwheat domestication. Moreover, the resistant haplotype was almost completely absent from the SL and NL groups (Fig. 3i; Additional file 2: Fig. S12). Subcellular localization experiments demonstrated that *FtGULO* was located in both the nucleus and cytoplasm (Fig. 3j), while the expression of *FtGULO* was induced by *R. solani* infection and methyl jasmonate (MeJA) treatment (Fig. 3k, l), suggesting *FtGULO* might be involved in jasmonate-mediated disease responses. Heterologous expression of *FtGULO* in *Arabidopsis* (Additional file 2: Fig. S13) demonstrated that the three *FtGULO* overexpression lines exhibited enhanced disease resistance compared to the wild type (Fig. 3m, n). In summary, these results illustrate that the natural variation in the promoter of *FtGULO* was involved in disease resistance reduction during Tartary buckwheat domestication through regulating *FtGULO* expression.

### Selection targets during Ecogeographic adaptation in China

**Fig. 4:**
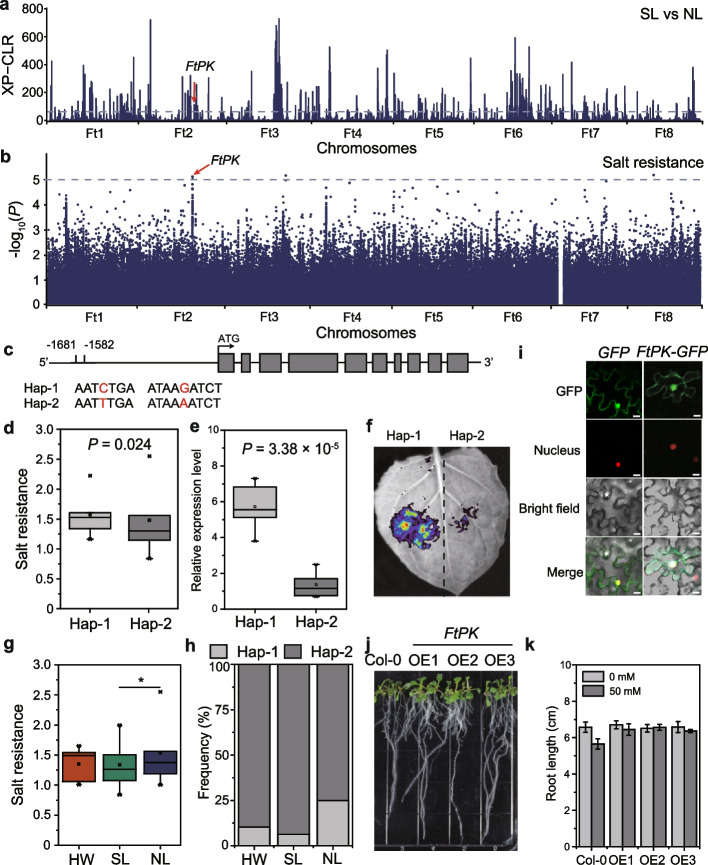
Variation of *FtPK* controls salt resistance differences between north and south populations of Tartary buckwheat. **A** Selective sweeps identified through comparisons between SL and NL using XP-CLR (cross-population composite likelihood-ratio test). The dashed line represents the top 5% of values therefore scores in these regions were regarded as selective sweeps. **B** Manhattan plot of GWAS signals for salt resistance in Tartary buckwheat accessions. The dashed line represents the threshold (-log_10_*P*=5). **C** Schematic diagram of *FtPK* gene structure. Two SNPs in the promoter of *FtPK* are marked with red letters and result in haplotypes (Hap) 1 and 2. **D** Box plots show salt resistance in two haplotypes (Hap). *n*_Hap-1_ = 13, *n*_Hap-2_ = 120. *P* value was calculated using two-tailed *t*-tests. **E** The expression level of *FtPK* in accessions with the two haplotypes. The error bars indicate the ± s. d, *n* = 6. The *P* value was calculated using one-way ANOVA. (**F**) Transcription activity of *FtPK* promoters with two haplotypes. **G** Differentiation salt resistance of accessions among HW, NL and SL groups. *n*_HW_ = 7, *n*_NL_ = 93, *n*_SL_ = 51. Significant differences were tested using two-tailed *t*-tests. *, *P* < 0.05. **H** Frequencies of the two haplotypes in the HW, NL and SL groups. **I** Confocal microscope image showing nuclear localization of FtPK-GFP fusion protein upon transient expression in *N. benthamiana* leaf cells. Scale bars, 10 µm. **J** Phenotypes of *Arabidopsis* lines heterologously expressing *FtPK* and subjected to salt stress. **K** Root length of *Arabidopsis* lines heterologously expressing *FtPK* and subjected to salt stress. Significant differences were tested using two-way ANOVA with Tukey HSD test. There was an effect of treatment (*F* = 11.044, df = 1, *P* = 0.004) and an effect of genotype (*F* = 4.478, df = 3, *P* = 0.018)

Environmental difference caused by varied geographical distribution are important reasons for crop divergence [33]. The northern and southern regions of China have highly different climates and soil, leading to the formation of locally adapted germplasm. To characterize the genetic basis of Tartary buckwheat differentiation caused by environmental adaptation, we used the XP-CLR and DCMS test to compare the SL and NL groups (Additional file 1: Table S13, S14). A total of 430 selective sweeps containing 2,968 genes were found that showed evidence of selection (Fig. 4a). Among them, methyl-cpg-binding domain protein was responsible for Arabidopsis flowering time regulation [44], while histone deacetylase [45] and ABA 8' hydroxylase [46] were involved in plant response to drought stress, heat shock proteins [47] were involved in Arabidopsis heat tolerance. Gene ontology (GO) and KEGG analysis revealed enrichment of categories involved in hormone, chemical, and auxin response, suggesting that response to divergent environments played a significant role in the divergence and evolution of these two groups (Additional file 2: Fig. S14).

In the arid and semi-arid regions of northern China, due to the low precipitation and the high evaporation, salt dissolved in the water is prone to accumulate on the soil surface, resulting in higher salt content in the soil [48]. To study the molecular basis of Tartary buckwheat adaption to this soil salinity difference, salt tolerance of 151 Tartary buckwheat accessions was investigated (Additional file 1: Table S15). A genome wide association study (GWAS) with salt tolerance as the phenotype (Fig. 4b; Additional file 1: Table S16) identified a significant association on chromosome 2, which overlapped with a selective sweep identified in the SL-NL XP-CLR test. Haplotype analysis identified two variants in the promoter of a gene encoding a protein kinase (*FtPK*; *FtPinG0201884400*; Fig. 4c). Phylogenetic analysis demonstrates this gene is an orthologue of protein kinase in other species (Additional file 2: Fig. S15). Accessions with Hap-l exhibited greater salt tolerance and *FtPK* expression compared to that with Hap-2 (Fig. 4d, e), and the frequency of Hap-1 in high soil Electrical Conductivity (ECE) was higher than that in low soil ECE conditions (Additional file 2: Fig. S16), suggesting *FtPK* might play a positive role in Tartary buckwheat salt tolerance. Transient activation assays demonstrated that promoters with Hap-1 possess higher transcription activity compared to those with Hap-2, further confirming the natural variations in the promoter of *FtPK* were involved in Tartary buckwheat salt tolerance (Fig. 4f). The salt tolerance was significantly lower in the SL groups than the NL group (Fig. 4g), further confirming the differentiation of salt tolerance in populations located in northern and southern China. The frequency of Hap-1 was greater in NL than SL (Fig. 4h; Additional file 2: Fig. S17). Subcellular localization experiments demonstrated that *FtPK* was located in both the nucleus and cytoplasm (Fig. 4i). Heterologous expression of *FtPK* in *Arabidopsis* was carried out (Additional file 2: Fig. S18) and resultant transformants were subjected to salt tolerance assays. The transgenic plants exhibited no reduction in root growth under salt treatment, whereas the WT showed reduced root growth (Fig. 4j, k) and this was accompanied by reduced leaf MDA content and greater POD activity after being exposed to salt (Additional file 2: Fig. S19). Taken together, these results illustrated that *FtPK* played an essential role in the divergence of north and south populations of Tartary buckwheat and this was related to the soil salt concentration.

### Human selection of easily dehulled Tatary buckwheat

**Fig. 5:**
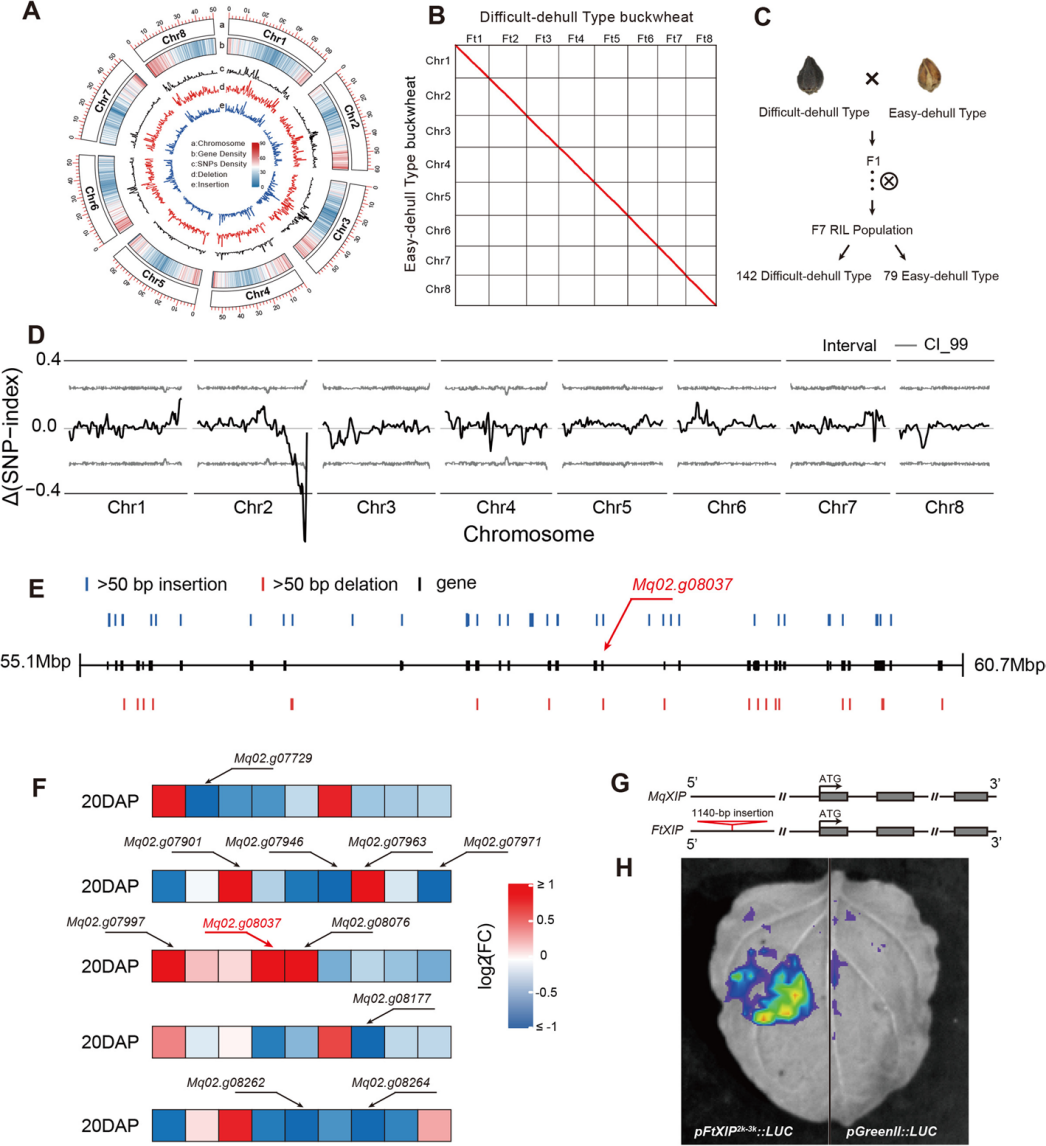
Structural variation of *FtXIP* controls the domestication of easily-dehulled type Tartary buckwheat. **A** Genome features of EDT. The outermost circle represents each chromosome of the genome. The second to fifth circles indicate gene density, SNPs density, deletion density, and insertion density, respectively, using a window size of 500-kb. **B** Gene dot map between easily-dehulled type buckwheat (EDT) and difficult-dehulled type (DDT) Tartary buckwheat. **C** Diagram representing the generation of the EDT x DDT recombinant inbred lines (RILs). **D** Genome wide Δ(SNP index) plot of the population derived from a cross between EDT and EDT. The black lines indicates tricube-smoothed Δ(SNP index), and the gray lines indicate corresponding two-sided 99% confidence intervals. **E** Insertions and deletions larger than 50 bp and within 5 kb of genes in the chr 2 QTL intervals. **F** Expression of genes with insertions and deletions in the QTL intervals in the seed coats of EDT and DDT at the 20-day after pollination (DAP) stage. Each small square represents the differentially expression level of a gene between EDT and DDT. Square with gene ID exhibited the differentially expressed genes. The red gene ID represents *FtXIP*. **G** Schematic diagram showing the deletion of 1,140 bp in the promoter region of *MqXIP* gene. **H** Transient expression assay was conducted to compare the transcription activity of *MqXIP* and an empty vector. **I** The expression level of *XIP* in DDT and EDT Tartary buckwheat. The error bars indicate the ± s. d, *n* = 6. The *P* value was calculated using two-tailed *t*-tests. *P* < 0.05

A unique Tartary buckwheat landrace from the SL group, the easily-dehulled type buckwheat (EDT, accession ID is YN600), was selected for further analysis. EDT is a variety of Tartary buckwheat grown for brewing by the Wa people - an ethnic minority in south-west China, and is the only Tartary buckwheat landrace with an easily dehulled phenotype [49]. The easily dehulled type has significantly contributed to the overall agricultural production of the crop [50]. Phylogenetic and genetic structure analyses revealed that this EDT landrace is grouped in SL1, which exhibited lower genetic diversity and slower LD decay compared to SL2 (Fig. 1), suggesting the higher domestication degree of these accessions compared to the others. To investigate the genetic basis of the easily dehulled phenotype of EDT, PacBio HiFi and Hi-C sequencing were conducted, followed by *de novo* genome assembly. A total of 30.59 Gb PacBio long reads recovering a total of 1,837 contigs were obtained (Additional file 1: Table S17). The longest contig was approximately 62.59 Mb and the N50 was 46.86 Mb (Additional file 1: Table S18). The contig-level assembly was then anchored into eight pseudo-chromosomes using the Hi-C data (Fig. 5a; Additional file 2: Fig. S20). The genome assembly of EDT exhibited a total size of 463.07 Mb (Additional file 1: Table S18). The analysis of the genome using Benchmarking Universal Single Copy Orthologs (BUSCO) against the embryophyte odb10 database revealed the presence of 96.6% complete BUSCOs in the EDT genome assembly (Additional file 1: Table S19). The genome sequence of EDT was annotated with RNA sequencing data from different tissues, resulting in a total of 36,229 protein-coding genes (Additional file 1: Table S18). The assembled genome of EDT exhibited strong collinearity with the genetic map constructed *de novo* from the RIL population data and the previously assembled genomes (V2 and HERA versions; Additional file 2: Fig. S21), demonstrating the reliability of the assembled genome.

Utilizing the high-quality genome assembly of EDT, pairwise genome alignment was conducted with Pinku1, a difficult-to-dehull type (DDT) from the NL group. A total of 344,323 SNPs and 99,617 indels (<50 bp) were detected in this comparison (Additional file 1: Table S20). Among these variants, 1.76% are nonsynonymous, potentially affecting gene function. 17,373 structural variants (SVs) with a size of ≥ 50 bp, including 2,881 insertions, 1,477 deletions, three translocations, and 19 inversions were discovered (Fig. 5b; Additional file 1: Table S20; Additional file 2: Fig. S22). To more precisely identify genes responsible for the easily dehulled phenotype, a recombinant inbred line (RIL) population, derived from a cross between EDT and DDT buckwheat, was constructed and, along with the parental lines, subjected to Illumina HiSeq2500-based re-sequencing [51]. Among the 221 F7 lines, 79 lines were predominantly EDT, and the remaining 142 lines were predominantly DDT (Fig. 5c). Quantitative trait locus (QTL) analysis identified one major QTL controlling the easily dehulled phenotype on Chr2 (Fig. 5d), which was consistent with the region identified previously [51]. Analyzing the insertions and deletions > 50 bp within the QTL interval, 54 genes that exhibited structural variants within the 5-kb range upstream and downstream were identified (Fig. 5e).

Subsequently, expression of these genes in EDT and DDT seeds was quantified [52]. Eleven genes displayed > 2-fold expression differences between EDT and DDT at the 20-day after pollination (DAP) stage of seed development (Fig. 5f; Additional file 1: Table S21). By combining the gene function annotations, a gene encoding a xylanase inhibitor (*XIP, Mq02.g08037*) that suppresses xylan degradation in the plant cell wall [53] was identified which could plausibly contribute to the easily dehulled trait. Compared to DDT, EDT exhibited a 1,140 bp deletion in the region 3-kb upstream of the start codon of *Mq02.g08037* (Fig. 5g). A transient activation assay demonstrated that the 1,140 bp sequence in the promoter resulted in significantly higher activity compared to the empty vector (Fig. 5h), and this region exhibited many cis-acting elements (Additional file 1: Table S22). And the expression of *XIP* is higher in DDT compared to EDT, suggesting this region could significantly up-regulate gene expression in developing DDT seeds. Hence, we speculated that the SV in the promoter region may have resulted in reduced expression of *Mq02.g08037*, ultimately leading to the easily dehulled trait in EDT.

## Discussion

As human societies around the world transitioned to agriculture, crop plants began the long-term process of domestication [54]. The only food crop in the Polygonaceae family, buckwheat is thought to have had its origin in south-eastern China [14–17]. However, due to the limited sampling and methods, more molecular evidence is needed to confirm this hypothesis. Previously, we attempted to validate the center of origin of Tartary buckwheat [8], however, the wild resources of Tartary buckwheat are mainly distributed in high-altitude areas of the Himalayas, posing serious challenges for the acquisition of this wild material. Here, we obtained 19,321,018 SNP from the genome re-sequencing data of 567 Tartary buckwheat accessions collected from throughout the world. Both the sampling representativeness and the variations are greater than previous studies [14, 15]. We found the HW group (Himalayan accessions enriched) exhibited higher nucleotide diversity (*π*) and faster LD decay compared to SL group (Yunnan and Sichuan accessions enriched) and NL group (northern China accessions enriched), confirming that Tartary buckwheat indeed originate from the Himalayan region, which is different from the center of origin of other grain crops of the Poaceae. As one of the youngest and loftiest mountain chains in the world, the Himalayas has unique climatic environments caused by large altitude variations, resulting in abundant plant diversity [55]. Thus, the confirmation of the Himalayan origin of Tartary buckwheat not only helps to protect the genetic diversity in its center of origin, thus promoting the use of wild germplasm resources for molecular breeding, but also has unique significance for the development of agricultural civilization, the protection of the global plant diversity.

Human migration has changed the face of the world, including the appearance and distribution of crops [56]. Due to the excellent environment for Tartary buckwheat cultivation, the Yi people, an ethnic minority of southwestern China, were the earliest people planting Tartary buckwheat where it is traditionally regarded as a staple food [23]. According to the Yi language classic 'Southwest Yi Annals', the ancestors of the Yi people came from 'outside the yak field', suggesting that the Yi people migrated from the Himalayan region. According to pollen abundance of Tartary buckwheat, the ancestors of the Yi people began planting Tartary buckwheat about 4,000 years ago [23]. By analyzing the genetic relationships and the timing of divergence between modern groups, we found that Tartary buckwheat in the southwest region spread from the Himalayas around 3,000~4,000 years ago, in exact accordance with the migration of the Yi people. There is a custom that brides bring their own Tartary buckwheat seeds as a dowry to their new homes, when the Yi people get married, which may promote the spread of Tartary buckwheat. Linguistic evidence suggested that European Tartary buckwheat is closely related to the Mongols. According to 'The History of The Mongol Empire', Tartary buckwheat spread to Europe with the expansion of the Mongol Empire. European historical data shows that Tartary buckwheat was introduced into Europe in the Middle Ages [21, 22]. A close phylogenetic relationship was found between accessions from northern China and outside China, indicating that Tartary buckwheat was introduced to Europe potentially only once from northern China [8]. However, due to only a few accessions used in our analysis which came from outside China, this conclusion needs further verification. The predicted divergence time suggested Tartary buckwheat was introduced to Europe around 1,000 years ago, which closely mirrors the time of the Mongols westward expansion. These results are of great significance not only for genetic improvement of Tartary buckwheat, but also for the understanding of the development of human cultures. In addition, as phylogeny showed individuals distributed in Qinghai-Gansu province (G5) were closer to their ancestors than other individuals distributed in Inner Mongolia-Hebei province (G6) and Hunan-Hubei-Jiangxi province (G7), and D-statistics exhibited a week gene flow (Z < 3) from individuals distributed in Qinghai-Gansu province to that distributed in Inner Mongolia-Hebei province, implying gene transfer between individuals in Qinghai-Gansu and Inner Mongolia-Hebei province.

Compared to wild germplasm resources, domesticated crops usually exhibit increased yield, better taste, and a plant architecture more suitable for cultivation. However, resistance to biotic or abiotic stress is often decreased during domestication, resulting in vulnerability to diseases and extreme weather and as such bringing severe yield losses [57]. Previous research demonstrated disease resistance associated metabolites are reduced in content in domesticated Tartary buckwheat relative to the wild accessions [12]. Here, by identifying selective sweeps between domesticated groups and the wild group, candidate genes responsible for domestication and diversification were identified. By combining genome-wide association studies with disease index of Tartary buckwheat collected worldwide, transcriptomics of Tartary buckwheat response to *R. solani* infection and MeJA treatment, *FtGULO*, a gene involved in ascorbate biosynthesis [42] was found to be responsible for decreased disease resistance in domesticated Tartary buckwheat. Only 25% resistant haplotype were identified in HW group, which might be due to that it is a newly generated haplotype in HW group and has not yet introgression into the domesticated group. But this speculation needs to be proved by further study. The exploration of such domestication genes will help transform wild plants into cultivated crops in a relatively short time by precisely changing key genes of important domestication traits [58].

Different genetic adaptations drive the formation of different ecotypes, and there are significant differences in the precipitation and temperature between northern and southern China, resulting in higher soil salinity in northern China compared to southern China [48]. We provide multiple lines of evidence that the increased frequency of a haplotype of *FtPK* with high expression is responsible for the greater salt tolerance of Tartary buckwheat from northern China than those from southern China. This suggest that *FtPK* plays an essential role in salt tolerance, which is according to the function of its houmologous [59, 60]. Besides the natural environment, the cultural environment will also generate unique germplasm resources that adapt to the dietary habits of local people [1]. The easily-dehulled type Tartary buckwheat is a unique landrace used for steaming as a staple food, wine- and tea- making in areas settled by the Wa people. Its easily dehulled nature of EDT allows local Wa people to use ancient artificial wooden mortars and pestles to dehull Tartary buckwheat and steam together with rice as staple food to prevent lysine deficiency. Comparative genomics and QTL analyses identified a xylanase inhibitor, a gene inhibiting the degradation of xylan, the main component of hemi-cellulose [53], was involved in the easily-dehulled phenotype. Not only do the results of this study demonstrate the center of origin and domestication history of Tartary buckwheat but the identification of genes responsible for important traits to productivity and cultivation that differentiate the groups, therefore providing important tools for the genetic improvement of this important dual use food and medicinal crop.

## Conclusions

In conclusion, our genomic studies provide valuable insights into the domestication, dispersal, and diversification of Tartary buckwheat. Through the analysis of wild and domesticated germplasm, we have unraveled the complex evolutionary history of this crop. The identification of selective sweeps, population relationship, and genetic markers associated with traits like salt tolerance has shed light on how adaptive processes and cultivation practices have shaped Tartary buckwheat. Additionally, the discovery of candidate genes, such as *FtPK*, has highlighted the molecular mechanisms underlying important agronomic traits. Further research and genetic investigations are necessary to fully comprehend the complexities and dynamics of its evolutionary journey.

## Materials and methods

### Genome re-sequencing, SNP calling and population structure analysis

A total of 567 Tartary buckwheat accessions, including 501 cultivated accessions and 66 wild accessions, were used in this study. Among them, 474 accessions were collected from China, and 93 accessions were collected from the other 16 countries (Additional file 1: Table S1). 489 accessions were re-sequenced in previous research [8, 61], and 78 accessions were newly re-sequenced in this study. Genomic DNA was extracted using cetyltrimethylammonium bromide (CTAB) as previously described [8]. Genomes were re-sequenced using Illumina NovaSeq 6000 platform. Raw reads in fastq file were trimmed to remove poor quality bases and adapters using Trimmomatic v0.33 [62] based on the manufacturer’s adapter sequences. A total of 7.7 Tb of clean data (i.e., after removing adapters, reads containing poly-N, and low-quality reads) was obtained. Clean reads were then mapped to the reference genome of Tartary buckwheat variety Pinku1 [63] using BWA-MEM [64]. After sorting by samtools, duplicated reads were removed using MarkDuplicates in Picard v1.13 (http://broadinstitute.github.io/picard/). Average depth was ~27.5× and mapping rate > 90% for each Tartary buckwheat accession. SNPs and small indels (1–50 bp) were called using the GATK pipeline [65]. Variants were called using GATK HaplotypeCaller, and then a joint-genotyping analysis of the gVCFs was performed on all merged samples. SNPs were filtered based on parameters previously used [8]. Population genetic structure was analyzed using the program ADMIXTURE v1.23 [66] with the putative number of populations (K values) from two to six. A maximum likelihood-based phylogenetic tree analysis was performed using IQ-TREE v1.6.6 [67]. Principal component analysis (PCA) was performed as previously described [8]. The nucleotide diversity (*π*) was calculated using VCFtools in 20-kb sliding windows with a 10-kb step. The fixation statistics (*F*_ST_) between different populations were calculated using a set of Python scripts (https://github.com/simonhmartin/genomics_general/popgeneWindows.py) with the parameters set as -w 100000, -s 10000, -f haplo.

### Identification of selective sweeps

To detect putative selective sweeps among different groups, the cross-population composite likelihood ratio test was performed using XP-CLR v1.1 [68]. Genome regions with top 5% XP-CLR values were considered as selected regions. Four statistics including XP-CLR, *π*, Theta and Tajima D were combined into a single DCMS framework [69]. Genome regions with *P* < 0.05 were considered as selective regions.

### GWAS analysis

Only SNPs with MAF ≥ 0.01 [70–72] and missing rate ≤ 0.1 in a population were used for GWAS. Efficient Mixed-Model Association eXpedited program (EMMAx) was used for GWAS analysis [73]. The significance threshold was set at *P* = 1×10^−5^.

### Admixture graph modeling and introgression analysis

The SNP dataset was filtered using ‘-mac 1 -max-alleles 2’ in VCFtools [74] and ‘-indep-pairwise 50 5 0.3’ in plink [75], and the convert program from AdmixTools was used to produce eigenstrat format data files. In order to measure allele sharing of three or four sets of subpopulations and to report the |Z|-score between predicted and observed values, the *f*_3_ and *F*_ST_ statistics were computed using ADMIXTOOLS 2.0 (https://uqrmaie1.github.io/admixtools) [76]. A heuristic algorithm to iteratively fit increasingly complex models, qpbrute (https://github.com/ekirving/qpbrute) filtered 1,183 possible admixture graph models and recorded ten graphs that left no *f*_4_ outliers (|Z| < 3) [77]. qpBayes [77] was then used to test the best-fit graph and compute the marginal likelihood of models and their Bayes factors. Analysis using qpGraph to detect the demographic graphs, and the best fitting model (no *f*_4_ outliers, |z|>=3) was carried out to assess putative population relationship under potential admixture events.

To remove the confounding effect from unclear subpopulation classification, we tested refined populations with additional silhouette filtering (Silhouette score >0) according to the methods described previously [78]. After filtering out monomorphic SNPs and those with missing data (missing rate ≤ 0.01), gene flow between the five population were estimated using Treemix v1.13 [79]. To refine the introgressed genomic regions, f_dM_ statistics were calculated along the whole genome using python scripts (https://github.com/simonhmartin/genomics_general) with 50-kb sliding windows and a 50k step. Geographic subsets of accessions were clustered using latitude and longitude coordinates by the K-means cluster method [80] with range extension less than 5 radius. After the filtering of multidimensional scaling analysis and silhouette examine of pairwise identity-by-state (IBS) distance matrix, ten representative groups consisting of 239 accessions were selected based on distinct population classification and sample size. Then the f-statistics and D-statistics were implemented using software referred as above. For D-statistics, only |Z score| >3 were considered as significant [31, 33, 81, 82].

### Estimation of divergence time and demographic history

The split function in SMC++ [83] was used to estimate the divergence times and the effective population size among different subpopulations. For normalizing population size, we randomly selected ten different samples of each subpopulation per time and ran 20 repeats that covered all samples. The mutation rate was set as 7×10^-9^ per synonymous site for each generation, and split time was calculated using one generation per year.

### Genome assembly and comparative genome analysis

The easily-dehulled type (EDT) genomes was assembled using PacBio HiFi reads and the hifiasm [84] assembly method. The Hi-C data was mapped to the corresponding contigs using the Juicer v1.6.2 pipeline [85]. Primary scaffolds were constructed using 3D-DNA v180922 [86] with default parameters. The assembly was visually inspected and manually curated using Juicebox Assembly Tools v1.9.8 [87]. Another round of scaffolding was performed using 3D-DNA v180922 to generate the final pseudo-chromosomes. To assess the completeness of the assembled genome, Benchmarking Universal Single-Copy Orthologous gene analysis (BUSCO) [88] was conducted using the conserved genes of the Embryophyta_odb10 as a reference. The SyRI v1.1 [89] comparison tool was used to identify SNP and SV between EDT and DDT using minimap2 v2.17 [90]. Structural variants were divided into four types: insertion, deletion, inversion and translocation.

### The genetic basis of the easily-dehulled phenotype and candidate genes prediction

To identify candidate mutations associated with the easily dehulled trait, an F7 population was generated from a cross between EDT and DDT accessions. The RILs (Recombinant Inbred Lines) in the population were classified into two groups based on their hull phenotype: easily-dehulled type or difficult-dehulled type. To identify variants between the parental genomes, SNPs (Single Nucleotide Polymorphisms) were calculated using the R package QTLseqr [91], resulting in a ΔSNP index. Each RIL individual was subjected to re-sequencing, and subsequently, individuals of the same dehulled type were merged. The resulting vcf file used for QTLseq analysis included four SNP datasets: EDT, DDT, EDT-RIL, and DDT-RIL. The genomic regions with a ΔSNP index exceeding the 99% confidence interval were considered candidate regions. Genes within these regions are putatively associated with the easily dehulled trait.

### Dual-luciferase assay

In the dual-luciferase assay, the promoter constructions were inserted into the pGreenII 0800-LUC vector for analysis. The *Agrobacterium tumefaciens* GV3101 strains carrying the respective promoter constructs were cultured overnight at 28 °C. The cultures were then diluted to an OD600 of 0.6 using resuspension buffer containing 10 mM MgCl_2_, 10 mM MES, and 100 mM acetosyringone. Separate *Nicotiana benthamiana* leaves were injected with *A. tumefaciens* carrying the construct. The injected leaves were incubated in the dark for 1 day and then exposed to 2 days of light/dark cycles (23℃/22℃, 16 h day/8 h night), after which the injected leaves were detached and sprayed with a solution of 1 mM D-Luciferin sodium salt and 0.01% Triton X-100 in ddH_2_O. The luminescence of the luciferase activity in the infiltrated area was captured using LB983 Nightowl II.

### Real-time quantitative PCR (qRT-PCR)

Total RNA was isolated from plant material using a plant RNA extraction kit (Aidlab, Beijing, China). The extracted RNA was reverse transcribed into cDNA by TRUEscript RT MasterMix PCR (Aidlab, Beijing, China). Primer sequences are listed in Additional file 1: Table S23. BnActin/AtActin was used as the reference and SYBR Green (Takara, Kyoto, Japan) was used as the fluorochrome. The amplification reactions were performed using a Line Gene K thermal cycler (BioRad, USA) under standard conditions.

### Transgenic plant construction and phenotype assay in *Arabidopsis thaliana*

Total RNA was extracted by using an RNApre Pure Plant Plus kit (Tiangen, Beijing, China). First-strand cDNA was synthesized with a HiScript III RT SuperMix for qPCR (Vazyme, Nanjing, China). The coding sequence was cloned into pCAMBIA-1302. The *Arabidopsis* overexpression lines were conducted and generated by *A. tumefaciens* GV3101 mediated transformation [92]. Three biological replicates were used, and the experiments were performed three times. Primer sequences are given in Additional file 1: Table S23. All *Arabidopsis* genotypes were grown at 22 °C (day/night) under long-day conditions (16-h light/8-h dark). Disease index evaluation was conducted as previously described [7, 93]. Root length and physiological and biochemical assays of *Arabidopsis* were used to evaluate the salt tolerance of transgenic plants. The effect of NaCl on root length of *Arabidopsis* was studied. Five-day-old Col-0 and *FtPK* transgenic Arabidopsis seedlings were transferred to 1/2MS Agar medium containing 50 mM NaCl, and root length was measured and photographed after vertical culture for 7 days. The determination of malondialdehyde (MDA) content and peroxidase (POD) activity were performed according to methods described previously [94]. Three biological replicates were conducted and the experiments were performed three times. The phylogenetic tree of GULOs and PKs were conducted using MEGA X based on the neighbor-joining method [95, 96].

### Salt tolerance assay in Tartary buckwheat germplasm resources

To a petri dish covered with two layers of filter paper was added 5mL water and 20 seeds were evenly placed on the filter paper and cultured at 25 (±1) ℃ with 12 hours daylength. Experiments were repeated three times. The germination rate, germination index and membership function value were calculated according to methods illustrated in the previous research [97]. GWAS was performed using membership function value. The Electrical Conductivity (ECE) was searched in Harmonized World Soil Database v 1.2 (HWSD v1.2) based on the longitude and latitude information of the location where accession obtained. Accessions with ECE < 0.2 were regards as samples from low-salinity land, and those with ECE > 1.9 were regards as samples from high-salinity land.

### Subcellular Localization

Full-length cDNAs of *FtGULO* and *FtPK* were amplified (primer sequences in Additional file 1: Table S23) and inserted into the pCAMBIA1300-GFP vector. p2300-35s-H2B-mCherry was used as a nuclear marker. The plasmid was transferred into *N. benthamiana* leaves using *A. tumefaciens* GV3101-mediated transient infiltration [92]. Subcellular localization was observed using a laser scanning confocal microscope (Zeiss LSM900) with the wavelengths of 488 (excitation)/500 to 530 nm (emission) for GFP and 561 (excitation)/590 to 640 nm (emission) for mCherry.

### Supplementary Information

**Additional file 1:** **Table S1.** Summary of the 567 Tartary buckwheat accessions and 7 outgroup accessions used in this study with the mapping rate, depth and coverage. **Table S2.** Distribution of SNPs into various genomic regions in Tartary buckwheat. **Table S3.** Statistical differences between pairwise *F*_ST_ values between the different populations. **Table S4.** Genomic fragments with evidence for introgression based on F_d_ and F_dM_.**Table S5.** Interpopulation introgression based on D- statistics. **Table S6.** Putative selective sweeps between HW and SL based on XP-CLR analysis. **Table S7.** Putative selective sweeps between HW and NL based on XP-CLR analysis.**Table S8.** Genes in selective sweeps in both between HW and SL and between HW and NL based on XP-CLR analysis. **Table S9.** Putative selective sweeps between HW and SL based on DCMS analysis. **Table S10.** Putative selective sweeps between HW and NL based on DCMS analysis. **Table S11.** Genes in selective sweeps in both between HW and SL and between HW and NL based on DCMS analysis. **Table S12.** Candidate genes associated with disease index identified by GWAS with MAF >= 0.01. **Table S13.** Putative selective sweeps between SL and NL based on XP-CLR. **Table S14.** Putative selective sweeps between SL and NL based on DCMS. **Table S15.** Salt tolerance of Tartary buckwheat accessions. **Table S16.** Candidate genes associated with salt tolerance identified by GWAS. **Table S17.** Sequencing reads used for assembly of EDT. **Table S18.** Assembly statistics of the EDT genome. **Table S19.** BUSCO analysis of the EDT genome. **Table S20.** Genetic differences between EDT and DDT. **Table S21.** The expression of genes with insertions and deletions within the QTL inverval. **Table S22.** Summary of *FtXIP* insertion promoter *cis*-acting elements prediction in 1,400 bp region. **Table S23.** Primers used in this study.

**Additional file 2:** **Figure S1.** Nucleotide diversity of HW, SL1, SL2, NLI and NLO groups. **Figure S2.** *F*_ST_ between different groups. **Figure S3.** Assessment of graph model of Tartary buckwheat accessions. **Figure S4.** The range of estimated divergence times between the populations. **Figure S5.** Divergence time between HW and SL, HW and NL, SL1 and SL2, NLI and NLO groups predicted with SMC++. **Figure S6.** Individuals of ten mini-groups based on geographical distribution was carried out using silhouette scoring. **Figure S7.** Silhouette scores of individuals used for Treemix analysis was carried out using silhouette scoring. **Figure S8.** Evaluation of introgression components between different population. **Figure S9.** Gene ontology analysis and KEGG enrichment analysis of genes in selective sweeps identified in both HW vs. SL and HW vs. NL comparisons. **Figure S10.** Local XP-CLR plot of the locus *FtGULO* located. **Figure S11.** FtGULO phylogenetic based on the neighbor-joining method tree using full-length amino acid sequences of orthologues genes in Tartary buckwheat and other plants. **Figure S12.** Geographic distribution of the Hap-A and Hap-T Tartary buckwheat accessions. **Figure S13.** PCR analysis of *Arabidopsis* lines heterologously expressing *FtGULO*. **Figure S14.** Gene ontology analysis and KEGG enrichment analysis of gene in regions of selective sweeps between SL and NL. **Figure S15.** FtPK phylogenetic tree based on the neighbor-joining method tree using full-length amino acid sequences of orthologues genes in Tartary buckwheat and other plants. **Figure S16.** Frequencies of the two haplotypes in the low ECE and high ECE groups. **Figure S17. **Geographic distribution of the Hap-1 and Hap-2 Tartary buckwheat accessions. **Figure S18.** PCR analysis of* Arabidopsis* lines heterologously expressing *FtPK*. **Figure S19.** MDA content and POD activity in *Arabidopsis *heterologously expressing *FtPK* compared to WT with and without a salt treatment. **Figure S20.** Hi-C contact matrix of the high-quality chromosome-scale genome assembly of EDT. **Figure S21.** Collinearity among the assembly of EDT, the genetic map of the RIL population, the HERA version assembly (DDT genome used in this study) and the V2 version assembly of Tartary buckwheat variety Pinku1 reference genome. **Figure S22.** The distribution of deletions and insertions between EDT and DDT on eight chromosomes of Tartary buckwheat.

**Additional file 3.** Review history.

## Data Availability

The raw data of newly re-sequenced 78 Tartary buckwheat accessions and the newly assembled genome of easily-dehulled type buckwheat are available from Genome Sequence Archive (GSA) [98] database in National Genomics Data Center  [99], China National Center for Bioinformation (CNCB) / Beijing Institute of Genomics, Chinese Academy of Sciences that are publicly accessible at https://ngdc.cncb.ac.cn, under accession no. PRJCA020346 [100]. Previously published whole-genome resequencing data of 496 Tartary buckwheat accessions were downloaded from the NCBI database under the accession no. PRJNA600676 [101]. Previously published raw sequencing data of the two parents and the 221 RILs were downloaded from the GSA database in CNCB under accession no. PRJCA003285  [102] . The scripts were deposited at the GitHub [103] and Zenodo [104].

## References

[1] Liu X, Li L, Li Y (2022). Synergistic evolution theory of crop germplasm resources and cultural environments. In Chinese. J Plant Genet Resour.

[2] Chen YH, Gols R, Benrey B (2015). Crop domestication and its impact on naturally selected trophic interactions. Annu Rev Entomol.

[3] Huang X, Huang S, Han B, Li J (2022). The integrated genomics of crop domestication and breeding. Cell.

[4] Wang Z, Miao L, Chen Y, Peng H, Ni Z, Sun Q, Guo W (2023). Deciphering the evolution and complexity of wheat germplasm from a genomic perspective. J Genet Genomics.

[5] Huda MN, Lu S, Jahan T (2021). Treasure from garden: Bioactive compounds of buckwheat. Food Chem.

[6] Schenke D, Utami HP, Zhou Z (2019). Suppression of UV-B stress induced flavonoids by biotic stress: Is there reciprocal crosstalk? Plant Physiol. Biochem.

[7] He Y, Zhang K, Li S (2023). Multi-omics analysis reveals the molecular mechanisms underlying virulence in *Rhizoctonia* and jasmonic acid-mediated resistance in Tartary buckwheat (*Fagopyrum tataricum*). Plant Cell.

[8] Zhang K, He M, Fan Y (2021). Resequencing of global Tartary buckwheat accessions reveals multiple domestication events and key loci associated with agronomic traits. Genome Biol.

[9] Zhu F (2016). Chemical composition and health effects of Tartary buckwheat. Food Chem.

[10] Hunt HV, Shang X, Jones MK (2018). Buckwheat: a crop from outside the major Chinese domestication centres? A review of the archaeobotanical, palynological and genetic evidence. Veg Hist Archaeobot.

[11] Alseekh S, Scossa F, Wen W (2021). Domestication of crop metabolomes: Desired and unintended consequences. Trends Plant Sci.

[12] Zhao H, He Y, Zhang K (2023). Rewiring of the seed metabolome during Tartary buckwheat domestication. Plant Biotechnol J.

[13] De Candolle, A. (1883). Origine des Plantes Cultivées; G. Baillière et cie: Paris, France, Volume 43.

[14] Tsuji K, Ohnishi O (2001). Phylogenetic relationships among wild and cultivated Tartary buckwheat (*Fagopyrum tataricum* Gaert.) populations revealed by AFLP analyses. Genes Genet Syst.

[15] Ohnishi O (1998). Search for the wild ancestor of buckwheat III. The wild ancestor of cultivated common buckwheat, and of Tatary buckwheat. Econ Bot.

[16] Ohnishi O, Konishi T (2001). Cultivated and wild buckwheat species in eastern Tibet. Fagopyrum.

[17] Fan Y, Ding M, Zhang K (2020). Overview and utilization of wild germplasm resources of the genus *Fagopyrum* Mill. In Chinese. J Plant Genet Resour.

[18] Bradley D (2011). Proto-Tibeto-Burman grain crops. Rice.

[19] Weisskopf A, Fuller DQ, Smith Claire (2014). Buckwheat: origins and development. Encyclopedia of Global Archaeology.

[20] Tang, Y., Ding, M., Tang, Y., et al. Germplasm resources of buckwheat in China. In Molecular Breeding and Nutritional Aspects of Buckwheat, Meiliang Zhou et al., ed. (Academic Press), 2016. pp. 13–20.

[21] Boivin N, Fuller DQ, Crowther A (2012). Old world globalization and the columbian exchange: comparison and contrast. World Archaeol.

[22] Hughes, D.H., and Henson, R.E. Crop production principles and practices. (The Macmillan Company). 1934.

[23] Yao YF, Song XY, Xie G (2023). New insights into the origin of buckwheat cultivation in southwestern China from pollen data. New Phytol.

[24] Smith BD (2001). Documenting plant domestication: The consilience of biological and archaeological approaches. Proc Natl Acad Sci USA.

[25] Zeder MA, Emshwiller E, Smith BD, Bradley DG (2006). Documenting domestication: the intersection of genetics and archaeology. Trends Genet.

[26] Huang X, Kurata N, Wei X (2012). A map of rice genome variation reveals the origin of cultivated rice. Nature.

[27] van Andel TR, Meyer RS, Aflitos SA (2016). Tracing ancestor rice of Suriname Maroons back to its African origin. Nat Plants.

[28] Wang W, Mauleon R, Hu Z (2018). Genomic variation in 3,010 diverse accessions of Asian cultivated rice. Nature.

[29] Hufford MB, Xu X, van Heerwaarden J (2012). Comparative population genomics of maize domestication and improvement. Nat Genet.

[30] Chen L, Luo J, Jin M (2022). Genome sequencing reveals evidence of adaptive variation in the genus *Zea*. Nat. Genet..

[31] Kang L, Qian L, Zheng M (2021). Genomic insights into the origin, domestication and diversification of *Brassica juncea*. Nat Genet.

[32] Wei T, van Treuren R, Liu X (2021). Whole-genome resequencing of 445 *Lactuca* accessions reveals the domestication history of cultivated lettuce. Nat Genet.

[33] Dong Y, Duan S, Xia Q (2023). Dual domestications and origin of traits in grapevine evolution. Science.

[34] Bellucci E, Benazzo A, Xu C (2023). Selection and adaptive introgression guided the complex evolutionary history of the European common bean. Nat Commun.

[35] Varshney RK, Roorkiwal M, Sun S (2021). A chickpea genetic variation map based on the sequencing of 3,366 genomes. Nature.

[36] Suarez-Gonzalez A, Lexer C, Cronk Q (2018). Adaptive introgression: a plant perspective. Biol Lett.

[37] Cai W, Hong J, Liu Z (2023). A receptor-like kinase controls the amplitude of secondary cell wall synthesis in rice. Curr Biol.

[38] Wang P (2022). Battle for survival: the role of plant thioredoxin in the war against *Barley stripe mosaic virus*. Plant Physiol.

[39] Breen S, Williams SJ, Outram M (2017). Emerging insights into the functions of pathogenesis-related protein 1. Trends Plant Sci.

[40] Cheng GY, Yang ZT, Zhang H (2020). Remorin interacting with PCaP1 impairs *Turnip mosaic virus* intercellular movement but is antagonised by VPg. New Phytol.

[41] Ji C, Xu L, Li Y (2022). The O2-ZmGRAS11 transcriptional regulatory network orchestrates the coordination of endosperm cell expansion and grain filling in maize. Mol Plant.

[42] Botanga CJ, Bethke G, Chen Z (2012). Metabolite profiling of *Arabidopsis* inoculated with *Alternaria brassicicola* reveals that ascorbate reduces disease severity. Mol Plant Microbe Interact.

[43] Paciolla C, Fortunato S, Dipierro N (2019). Vitamin C in plants: From functions to biofortification. Antioxidants.

[44] Potok ME, Wang YF, Xu LH (2019). Arabidopsis SWR1-associated protein methyl-CpG-binding domain 9 is required for histone H2A.Z deposition. Nat Commun.

[45] Baek D, Shin G, Kim MC (2020). Histone deacetylase HDA9 with ABI4 contributes to abscisic acid homeostasis in drought stress response. Front Plant Sci.

[46] Blankenagel S, Eggels S, Frey M (2022). Natural alleles of the abscisic acid catabolism gene *ZmAbh4* modulate water use efficiency and carbon isotope discrimination in maize. Plant Cell.

[47] Jacob P, Hirt H, Bendahmane A (2017). The heat-shock protein/chaperone network and multiple stress resistance. Plant Biotechnol J.

[48] Liu L, Wang B (2021). Protection of halophytes and their uses for cultivation of saline-alkali soil in China. Biology (Basel).

[49] Campbell C (2003). Buckwheat crop improvement. Fagopyrum.

[50] Peng Y, Yan H, Guo L (2022). Reference genome assemblies reveal the origin and evolution of allohexaploid oat. Nat Genet.

[51] Shi TX, Li RY, Zheng R (2021). Mapping QTLs for 1000-grain weight and genes controlling hull type using SNP marker in Tartary buckwheat (*Fagopyrum tataricum*). BMC Genomics.

[52] Li HY, Wu CX, Lv QY (2020). Comparative cellular, physiological and transcriptome analyses reveal the potential easy dehulling mechanism of rice-tartary buckwheat (*Fagopyrum tararicum*). BMC Plant Biol.

[53] Fierens E, Rombouts S, Gebruers K (2007). TLXI, a novel type of xylanase inhibitor from wheat (*Triticum aestivum*) belonging to the thaumatin family. Biochem J.

[54] Meyer RS, DuVal AE, Jensen HR (2012). Patterns and processes in crop domestication: an historical review and quantitative analysis of 203 global food crops. New Phytol.

[55] Pandit MK (2020). The Himalaya should be a nature reserve. Nature.

[56] Ning C, Li T, Wang K (2020). Ancient genomes from northern China suggest links between subsistence changes and human migration. Nat. Commun.

[57] Zhu G, Wang S, Huang Z (2018). Rewiring of the fruit metabolome in tomato breeding. Cell.

[58] Gasparini K, Moreira JDR, Peres LEP, Zsögön A (2021). *De novo* domestication of wild species to create crops with increased resilience and nutritional value. Curr Opin Plant Biol.

[59] Zhu JK (2002). Salt and drought stress signal transduction in plants. Annu Rev Plant Biol.

[60] Zhou H, Shi H, Yang Y, Feng X, Chen X, Xiao F, Lin H, Guo Y (2023). Insights into plant salt stress signaling and tolerance. J Genet Genomics.

[61] Lai D, Zhang K, He Y (2023). Multi-omics identification of a key glycosyl hydrolase gene *FtGH1* involved in rutin hydrolysis in Tartary buckwheat (*Fagopyrum tataricum*). Plant Biotechnol J.

[62] Bolger AM, Lohse M, Usadel B (2014). Trimmomatic: a flexible trimmer for Illumina sequence data. Bioinformatics.

[63] Du H, Liang C (2019). Assembly of chromosome-scale contigs by efficiently resolving repetitive sequences with long reads. Nat Commun.

[64] Li, H. Aligning sequence reads, clone sequences and assembly contigs with BWA-MEM. arXiv. preprint arXiv: 2013; 1303: 997.

[65] McKenna A, Hanna M, Banks E (2010). The genome analysis toolkit: a MapReduce framework for analyzing next-generation DNA sequencing data. Genome Res.

[66] Alexander DH, Novembre J, Lange K (2009). Fast model-based estimation of ancestry in unrelated individuals. Genome Res.

[67] Nguyen LT, Schmidt HA, von Haeseler A, Minh BQ (2015). IQ-TREE: a fast and effective stochastic algorithm for estimating maximum-likelihood phylogenies. Mol Biol Evol.

[68] Chen H, Patterson N, Reich D (2010). Population differentiation as a test for selective sweeps. Genome Res.

[69] Ma Y, Ding X, Qanbari S, Weigend S, Zhang Q, Simianer H (2015). Properties of different selection signature statistics and a new strategy for combining them. Heredity (Edinb).

[70] Li N, Zhang X, Sun X (2022). Genomic insights into the evolutionary history and diversification of bulb traits in garlic. Genome Biol.

[71] Li N, He Q, Wang J (2023). Super-pangenome analyses highlight genomic diversity and structural variation across wild and cultivated tomato species. Nat Genet.

[72] Zhang H, Mi S, Brito L, Hu L, Wang L, Ma L, Xu Q, Guo G, Yu Y, Wang Y (2023). Genomic and transcriptomic analyses enable the identification of important genes associated with subcutaneous fat deposition in Holstein cows. J Genet Genomics.

[73] Kang HM, Sul JH, Service SK (2010). Variance component model to account for sample structure in genome-wide association studies. Nat Genet.

[74] Danecek P, Auton A, Abecasis G (2011). The variant call format and VCFtools. Bioinformatics.

[75] Purcell S, Neale B, Todd-Brown K (2007). PLINK: A tool set for whole-genome association and population-based linkage analyses. Am J Hum Genet.

[76] Patterson N, Moorjani P, Luo Y (2012). Ancient admixture in human history. Genetics.

[77] Feuerborn TR, Carmagnini A, Losey RJ (2021). Modern Siberian dog ancestry was shaped by several thousand years of Eurasian-wide trade and human dispersal. Proc Natl Acad Sci USA.

[78] Swarts K, Gutaker RM, Benz B (2017). Genomic estimation of complex traits reveals ancient maize adaptation to temperate North America. Science.

[79] Fitak RR (2021). OptM: estimating the optimal number of migration edges on population trees using Treemix. Biol Methods Protoc.

[80] Macqueen, J. Some methods for classification and analysis of multivariate observations. Proc. Symp. Math. Statist. and Probability, 5th, 1. 1967.

[81] Durand EY, Patterson N, Reich D, Slatkin M (2011). Testing for ancient admixture between closely related populations. Mol Biol Evol.

[82] Villa-Islas V, Izarraras-Gomez A, Larena M (2023). Demographic history and genetic structure in pre-Hispanic Central Mexico. Science.

[83] Terhorst J, Kamm JA, Song YS (2017). Robust and scalable inference of population history from hundreds of unphased whole genomes. Nat Genet.

[84] Feng X, Cheng H, Portik D, Li H (2022). Metagenome assembly of high-fidelity long reads with hifiasm-meta. Nat Methods.

[85] Durand NC, Shamim MS, Machol I (2016). Juicer provides a One-Click system for analyzing Loop-Resolution Hi-C experiments. Cell Syst.

[86] Dudchenko O, Batra SS, Omer AD (2017). De novo assembly of the *Aedes aegypti* genome using Hi-C yields chromosome-length scaffolds. Science.

[87] Durand NC, Robinson JT, Shamim MS (2016). Juicebox provides a visualization system for Hi-C contact maps with unlimited zoom. Cell Syst.

[88] Simao FA, Waterhouse RM, Ioannidis P (2015). BUSCO: assessing genome assembly and annotation completeness with single-copy orthologs. Bioinformatics.

[89] Goel M, Sun H, Jiao WB, Schneeberger K (2019). SyRI: finding genomic rearrangements and local sequence differences from whole-genome assemblies. Genome Biol.

[90] Li H (2018). Minimap2: pairwise alignment for nucleotide sequences. Bioinformatics.

[91] Mansfeld BN, Grumet R (2018). QTLseqr: An R package for bulk segregant analysis with next-generation sequencing. Plant Genome.

[92] Wang X, Prokhnevsky AI, Skarjinskaia M (2023). Facilitating viral vector movement enhances heterologous protein production in an established plant system. Plant Biotechnol J.

[93] Park DS, Sayler RJ, Hong YG, Nam MH, Yang Y (2008). A method for inoculation and evaluation of rice sheath blight disease. Plant Dis..

[94] Niu C, Jiang L, Cao F (2022). Methylation of a MITE insertion in the *MdRFNR1-1* promoter is positively associated with its allelic expression in apple in response to drought stress. Plant Cell.

[95] Saitou N, Nei M (1987). The neighbor-joining method: a new method for reconstructing phylogenetic trees. Mol Biol Evol.

[96] Kumar S, Stecher G, Li M (2018). MEGA X: Molecular evolutionary genetics analysis across computing platforms. Mol Biol Evol.

[97] Yan S, Chong P, Zhao M (2022). Effect of salt stress on the photosynthetic characteristics and endogenous hormones, and: A comprehensive evaluation of salt tolerance in *Reaumuria soongorica* seedlings. Plant Signal Behav.

[98] Chen T, Chen X, Zhang S (2021). The genome sequence archive family: Toward explosive data growth and diverse data types. Genom Proteom Bioinform.

[99] CNCB-NGDC Members and Partners (2022). Database resources of the National Genomics Data Center, China National Center for Bioinformation in 2022. Nucleic Acids Res.

[100] He, Y., Zhang, K., Shi, Y., et al. Genomic insight into the origin, domestication, dispersal and diversification of Tartary buckwheat. PRJCA020346. Genome reseguencing of wild and foreign Tartary buckwheat. 2024. https://ngdc.cncb.ac.cn/bioproject/browse/PRJCA020346. 10.1186/s13059-024-03203-z PMC10898187 38414075

[101] Zhang, K., He, M., Fan, Y., et al. Resequencing of global Tartary buckwheat accessions reveals multiple domestication events and key loci associated with agronomic traits. PRJNA600676. *Fagopyrum tataricum* clean sequence reads. 2021. https://www.ncbi.nlm.nih.gov/bioproject/?term=PRJNA600676. 10.1186/s13059-020-02217-7 PMC7802136 33430931

[102] Shi, T., Li, R., Zheng, R., et al. Mapping QTLs for 1000-grain weight and genes controlling hull type using SNP marker in Tartary buckwheat (Fagopyrum tataricum). PRJCA003285. Construction Linkage Map of Tartary Buckwheat Based on RAD. 2021. https://ngdc.cncb.ac.cn/bioproject/browse/PRJCA003285. 10.1186/s12864-021-07449-w PMC7913328 33639857

[103] He, Y., Zhang, K., Shi, Y., et al. Genomic insight into the origin, domestication, dispersal and diversification of Tartary buckwheat. GitHub. 2024. https://github.com/Buckwheat-lab/Tartary_buckwheat_WGS. 10.1186/s13059-024-03203-z PMC10898187 38414075

[104] He Y, Zhang K, Shi Y, et al. Genomic insight into the origin, domestication, dispersal and diversification of Tartary buckwheat. 2024. Zenodo. 10.5281/zenodo.10663969. 10.1186/s13059-024-03203-z PMC10898187 38414075

