# Supplementary material for: Genomic insight into the origin, domestication, dispersal, diversification and human selection of Tartary buckwheat
Source: Genome Biol. 2024 Feb 27;25:61. doi: 10.1186/s13059-024-03203-z (PMC10898187; doi:10.1186/s13059-024-03203-z)
Supplement: Supplementary file 2 — Additional file 2: Figure S1. Nucleotide diversity of HW, SL1, SL2, NLI and NLO groups. Figure S2. FST between different groups. Figure S3. Assessment of graph model of Tartary buckwheat accessions. Figure S4. The range of estimated divergence times between the populations. Figure S5. Divergence time between HW and SL, HW and NL, SL1 and SL2, NLI and NLO groups predicted with SMC++. Figure S6. Individuals of ten mini-groups based on geographical distribution was carried out using silhouette scoring. Figure S7. Silhouette scores of individuals used for Treemix analysis was carried out using silhouette scoring. Figure S8. Evaluation of introgression components between different population. Figure S9. Gene ontology analysis and KEGG enrichment analysis of genes in selective sweeps identified in both HW vs. SL and HW vs. NL comparisons. Figure S10. Local XP-CLR plot of the locus FtGULO located. Figure S11. FtGULO phylogenetic based on the neighbor-joining method tree using full-length amino acid sequences of orthologues genes in Tartary buckwheat and other plants. Figure S12. Geographic distribution of the Hap-A and Hap-T Tartary buckwheat accessions. Figure S13. PCR analysis of Arabidopsis lines heterologously expressing FtGULO. Figure S14. Gene ontology analysis and KEGG enrichment analysis of gene in regions of selective sweeps between SL and NL. Figure S15. FtPK phylogenetic tree based on the neighbor-joining method tree using full-length amino acid sequences of orthologues genes in Tartary buckwheat and other plants. Figure S16. Frequencies of the two haplotypes in the low ECE and high ECE groups. Figure S17. Geographic distribution of the Hap-1 and Hap-2 Tartary buckwheat accessions. Figure S18. PCR analysis of Arabidopsis lines heterologously expressing FtPK. Figure S19. MDA content and POD activity in Arabidopsis heterologously expressing FtPK compared to WT with and without a salt treatment. Figure S20. Hi-C contact matrix of the high-quality chromosome-scale g [file 13059_2024_3203_MOESM2_ESM.pdf]

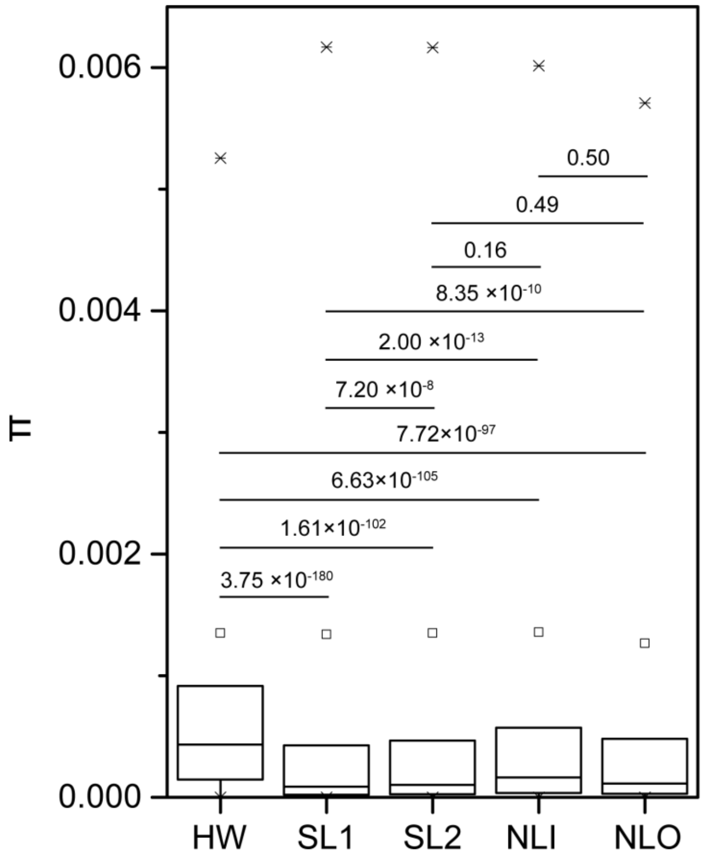

Figure S1. Nucleotide diversity (*π*) of HW, SL1, SL2, NLI and NLO groups. *P* values were calculated using two-tailed *t*-tests. The bottom and top of the box represent the first and third quartiles. The line is the median and the little squares extend to farthest data points within 1.5× interquartile range outside box edges. The crosses higher up represent outliers.

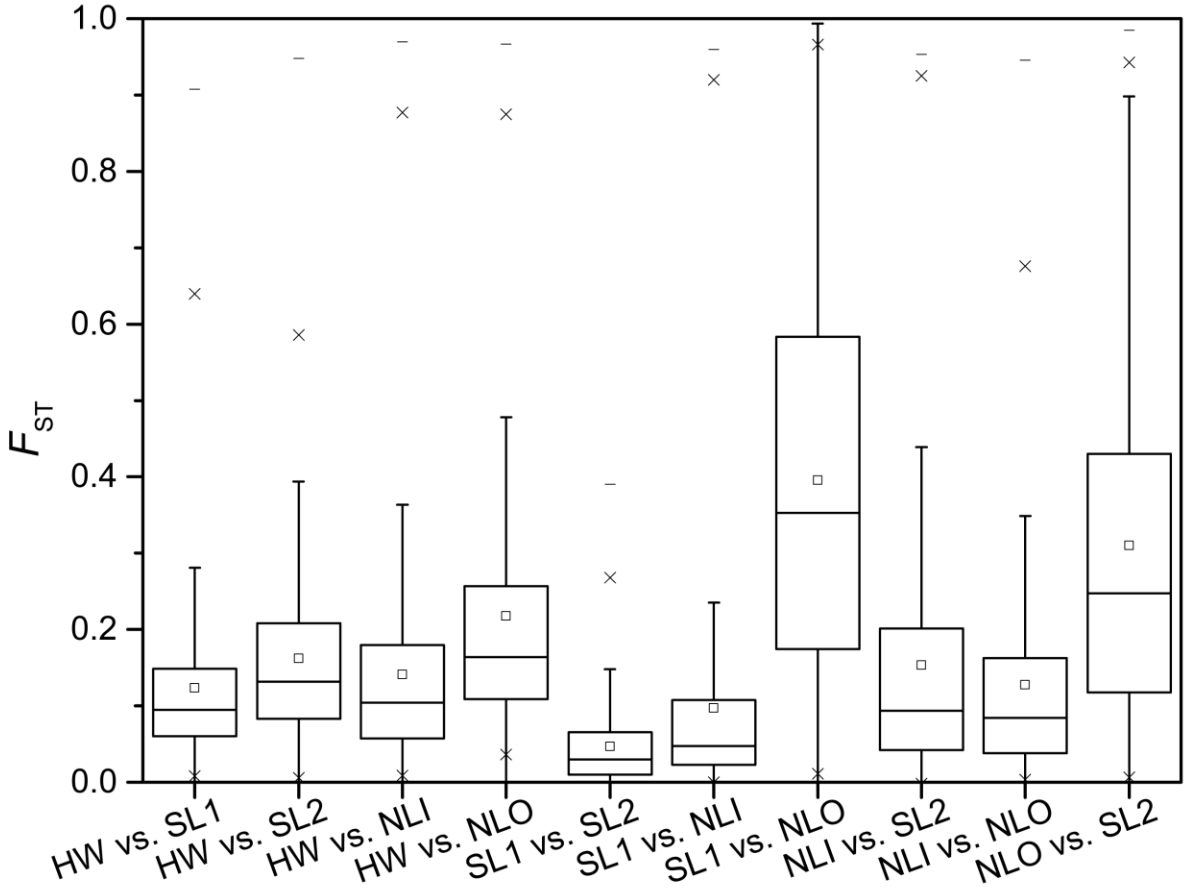

Figure S2. F_{ST} between different groups. The bottom and top of the box represent the first and third quartiles. The line is the median and the little squares extend to farthest data points within 1.5× interquartile range outside box edges. The crosses higher up represent outliers.

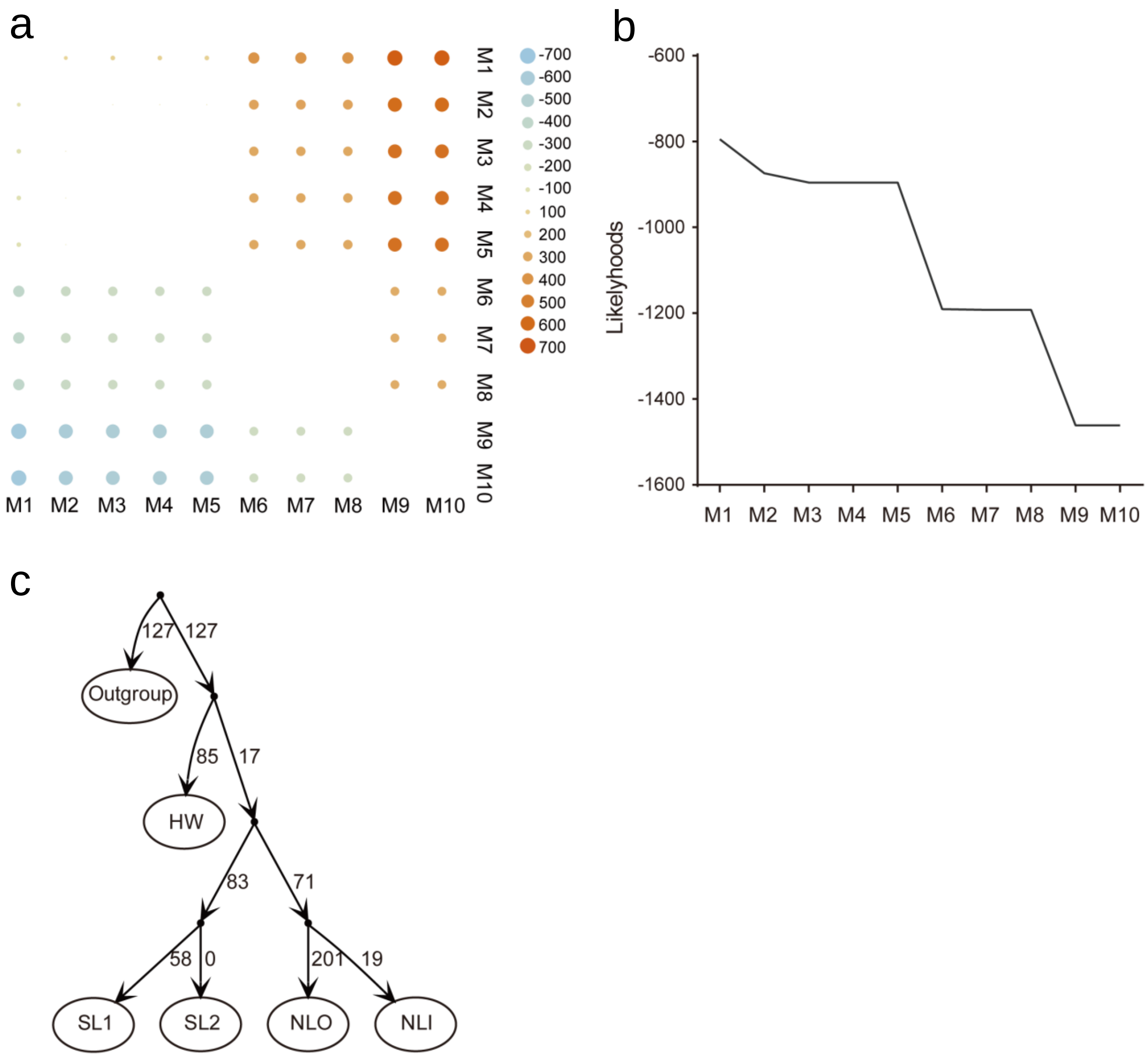

Figure S3. Assessment of graph model of Tartary buckwheat accessions. (a) Comparation of Bayes factors of 10 candidate models. (b) Likelihoods of models estimated by qpBayes. (c) Best fitting model tested by admixture graph.

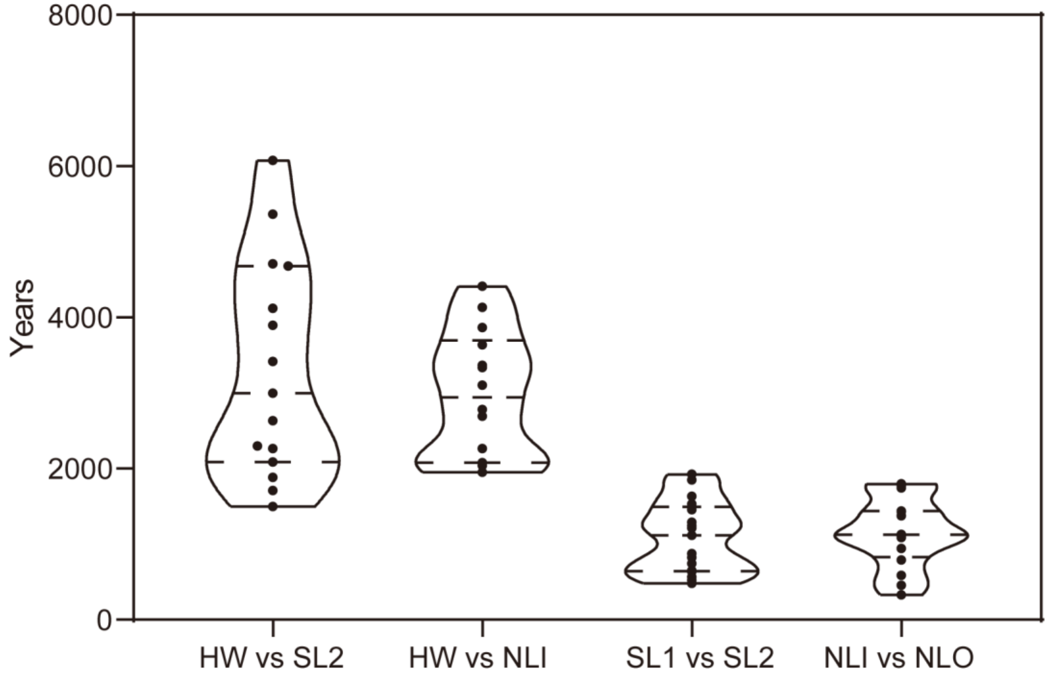

Figure S4. The range of estimated divergence times between the populations. The bottom and top of the violin represent the maximum and minimum values. The bold dash line is the median, and the dash lines represent the first and third quartiles. Points of different shapes represent the data distribution.

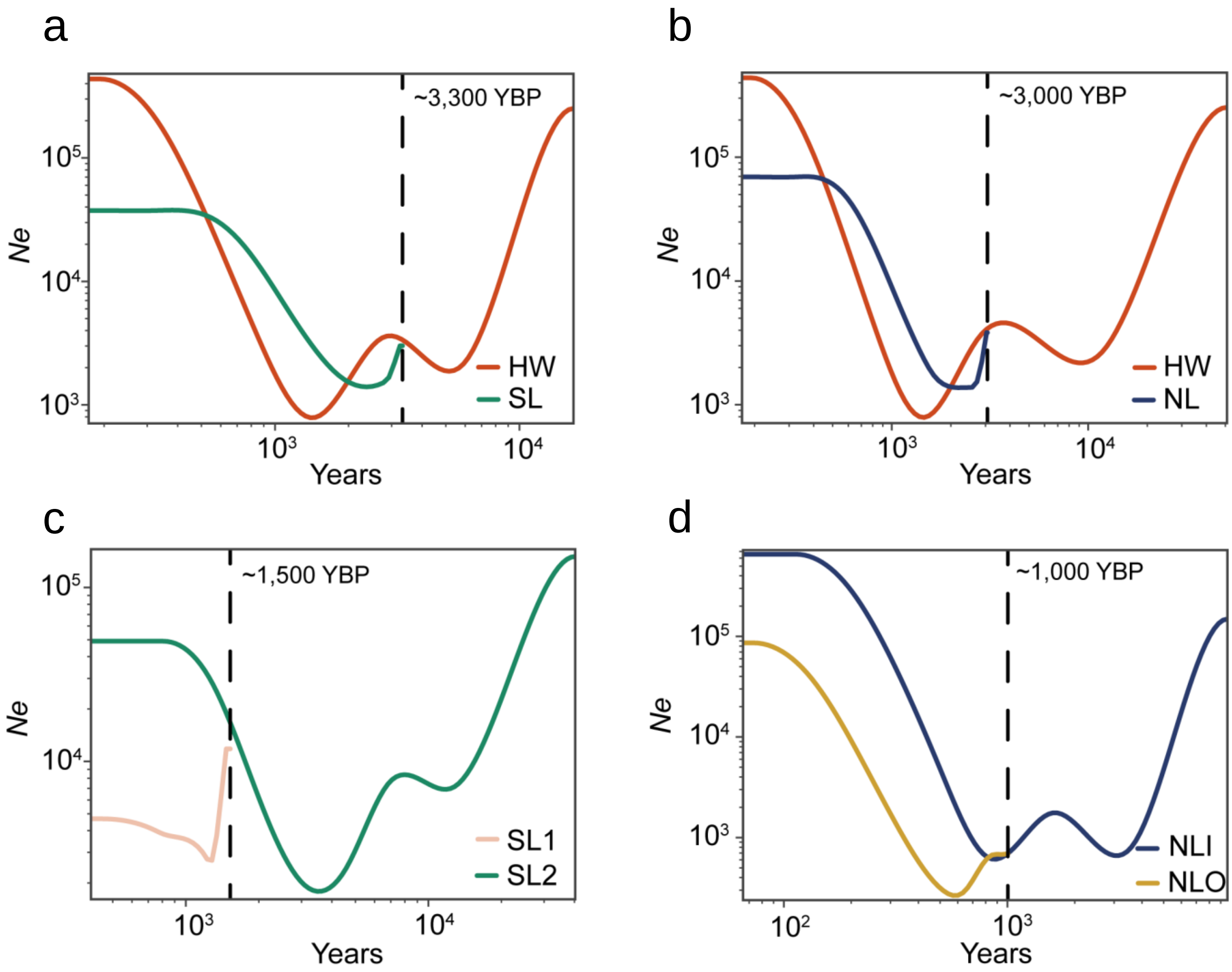

Figure S5. Divergence time between HW and SL (a), HW and NL (b), SL1 and SL2 (c), NLI and NLO (d) groups predicted with SMC++. Each splitting time between two groups was marked with black dashed line. YBP, years before present.

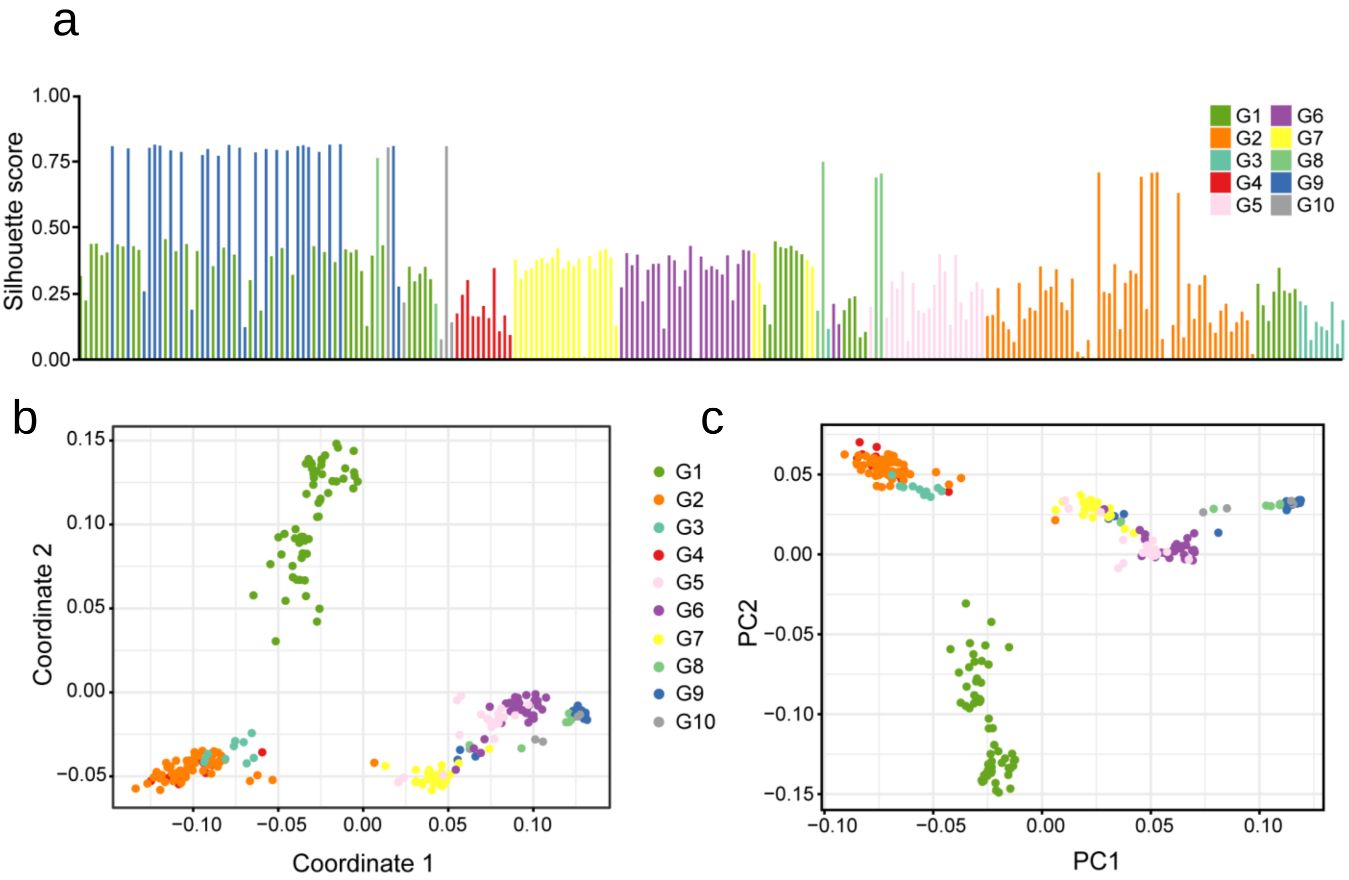

Figure S6. Individuals of ten mini-groups based on geographical distribution was carried out using silhouette scoring. (a) Silhouette scores of mini-groups. (b-c) MDS (b) and PCA (c) plots showing the cluster of ten groups.

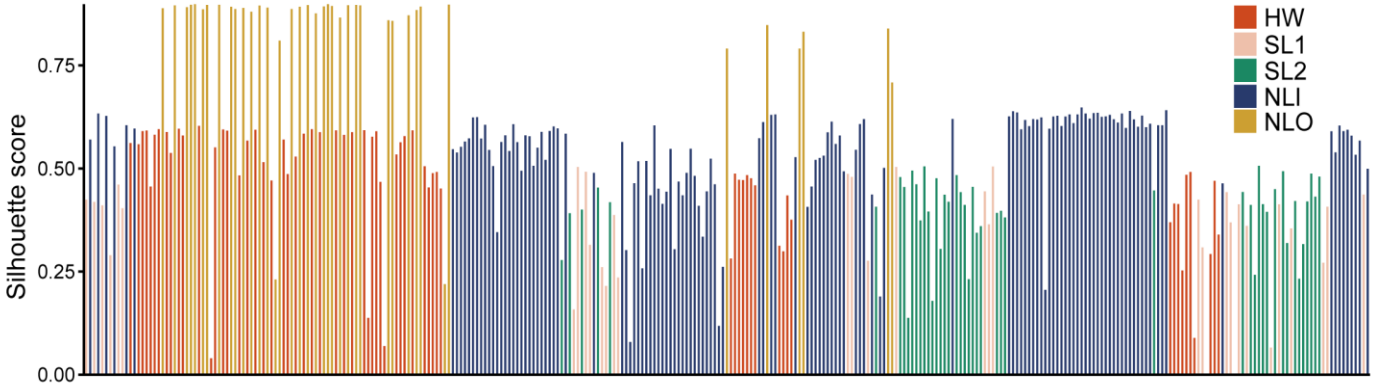

Figure S7. Silhouette scores of individuals used for Treemix analysis was carried out using silhouette scoring.

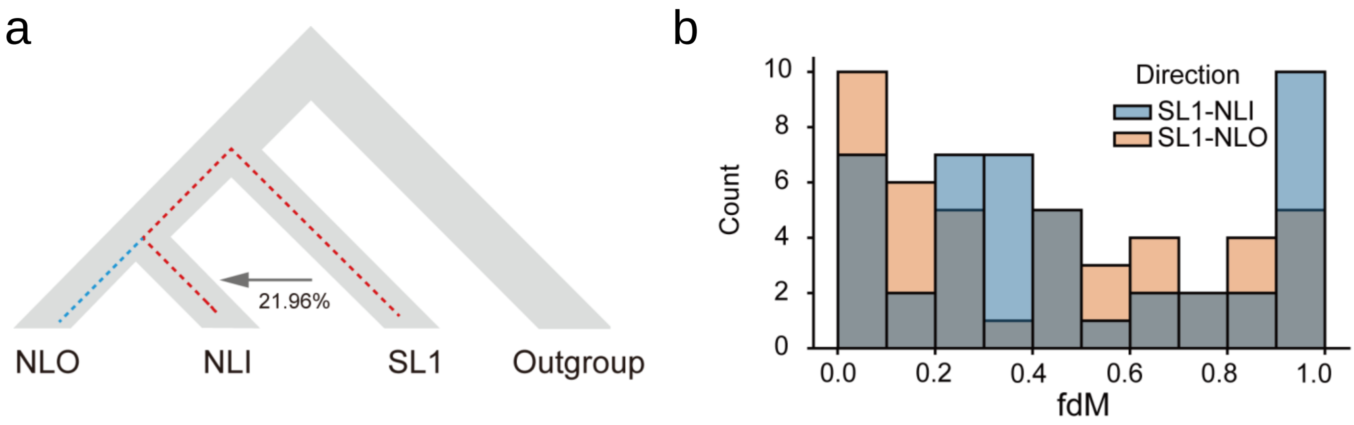

Figure S8. Evaluation of introgression components between different population. F_{dM} values (a) and fragment counts (b) from SL1 to NL population.

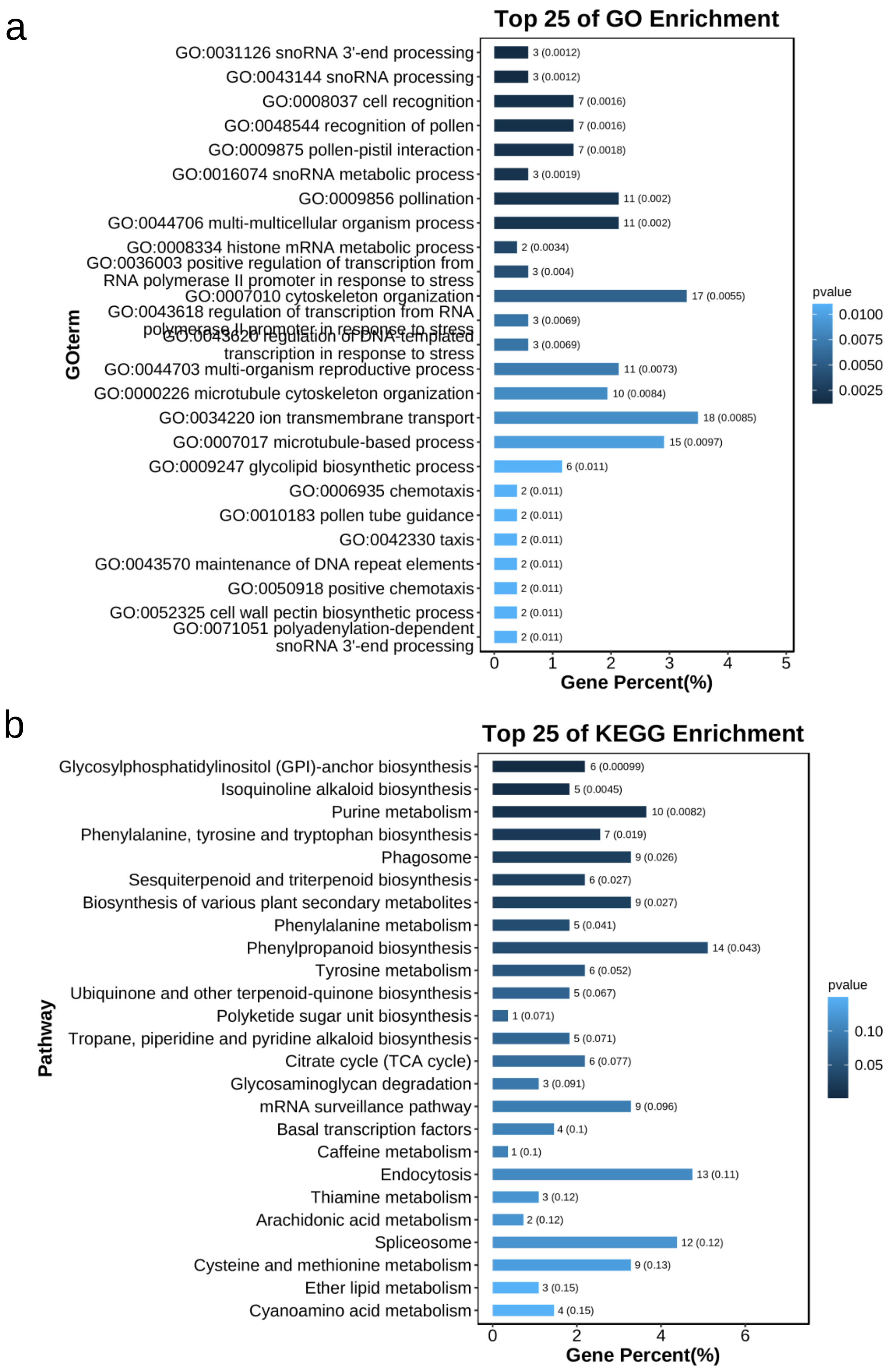

Figure S9. Gene ontology (GO) analysis (a) and KEGG enrichment analysis (b) of genes in selective sweeps identified in both HW vs. SL and HW vs. NL comparisons.

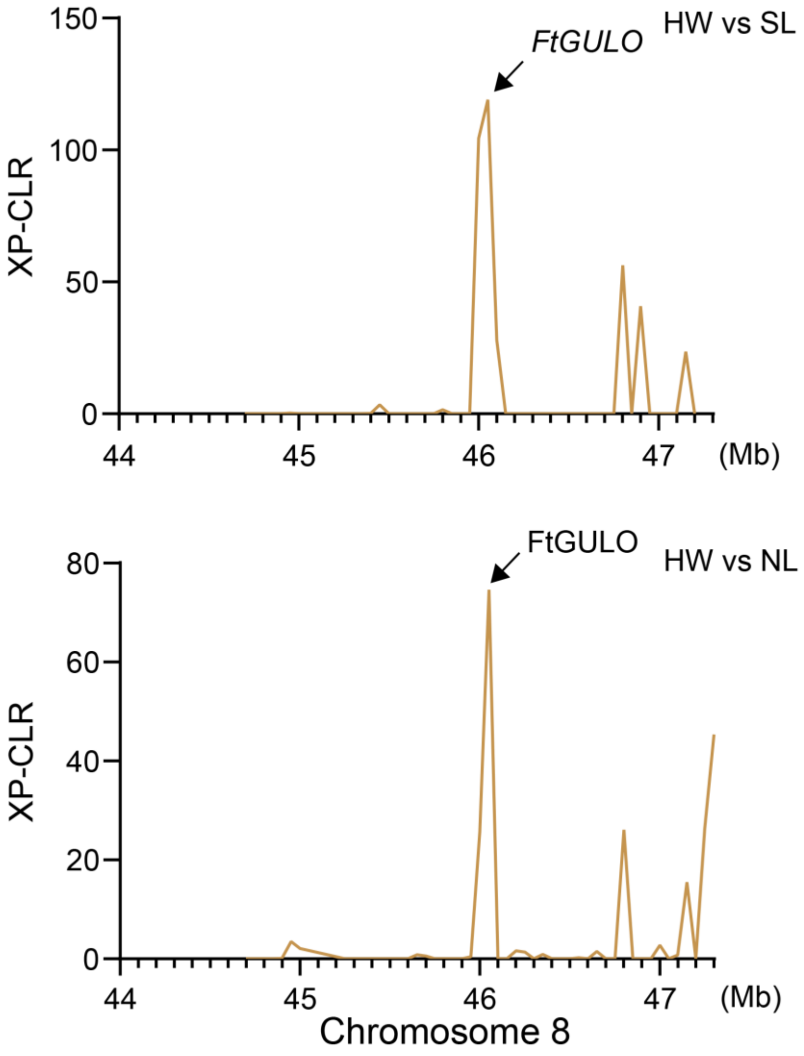

Figure S10. FtGULO phylogenetic based on the neighbor-joining method tree using full-length amino acid sequences of orthologues genes in Tartary buckwheat (*FtGULO*) and other plants.

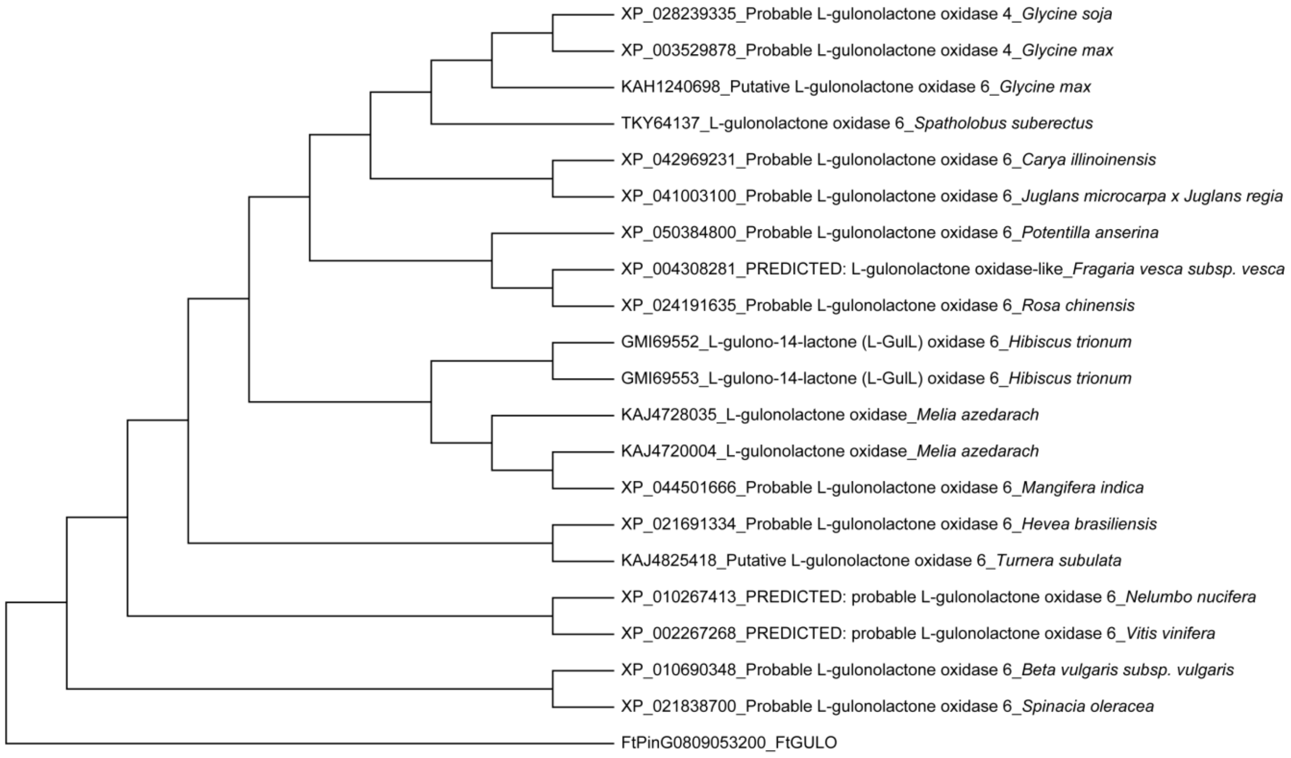

Figure S11. FtGULO phylogenetic based on the neighbor-joining method tree using full-length amino acid sequences of orthologues genes in Tartary buckwheat (*FtGULO*) and other plants.

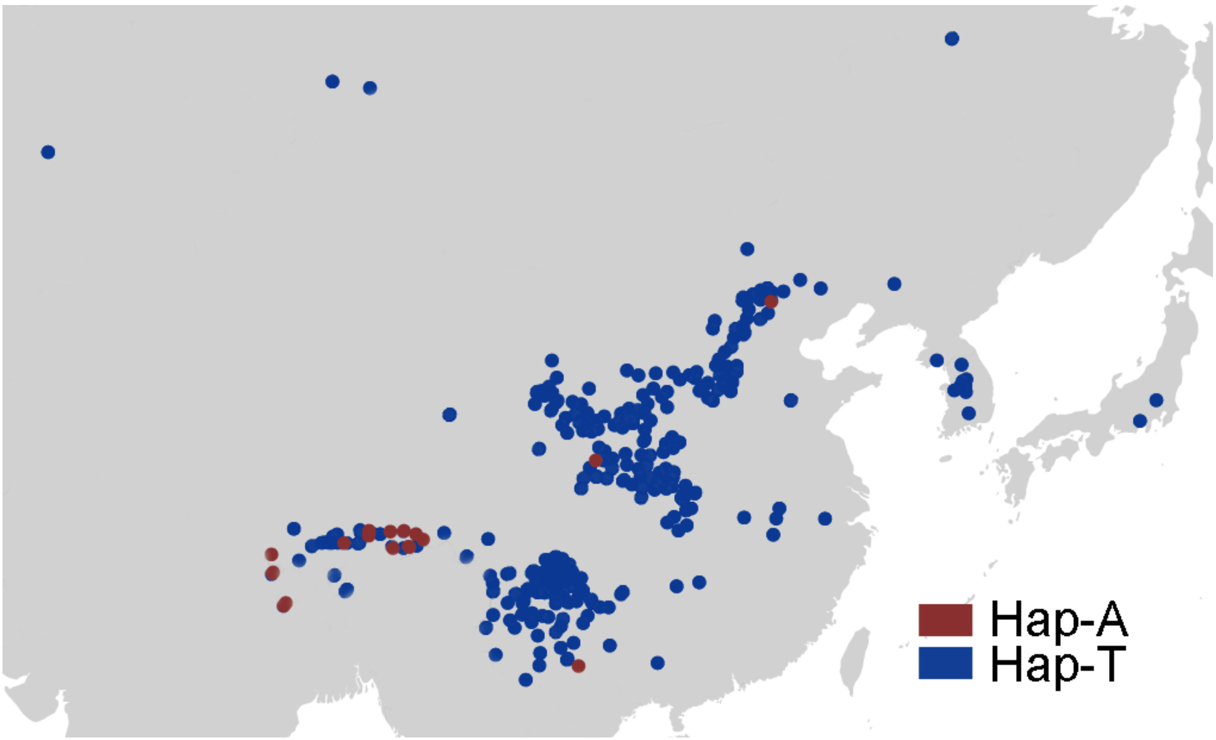

Figure S12. Geographic distribution of the Hap-A (red) and Hap-T (blue) Tartary buckwheat accessions.

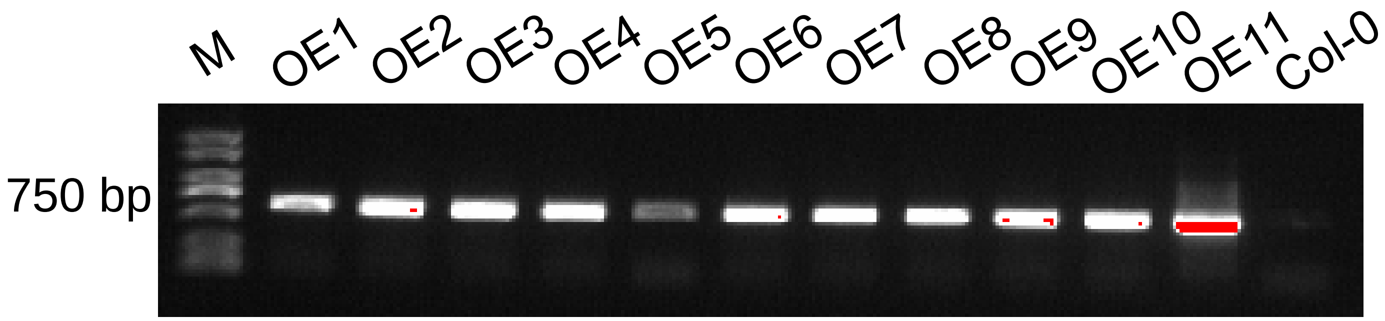

Figure S13. PCR analysis of *Arabidopsis* lines heterologously expressing *FtGULO*.

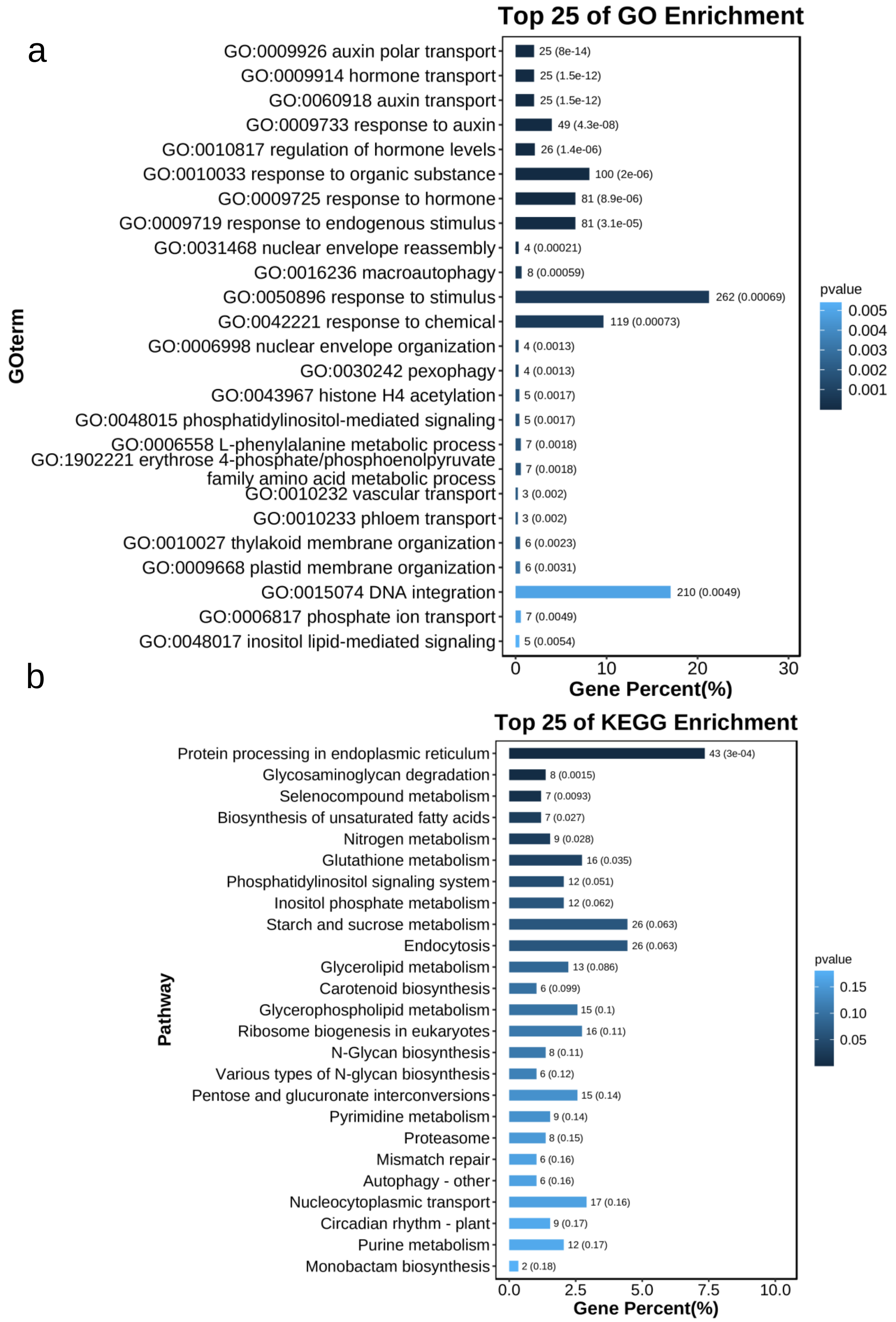

Figure S14. Gene ontology (GO) analysis (a) and KEGG enrichment analysis (b) of gene in regions of selective sweeps between SL and NL.

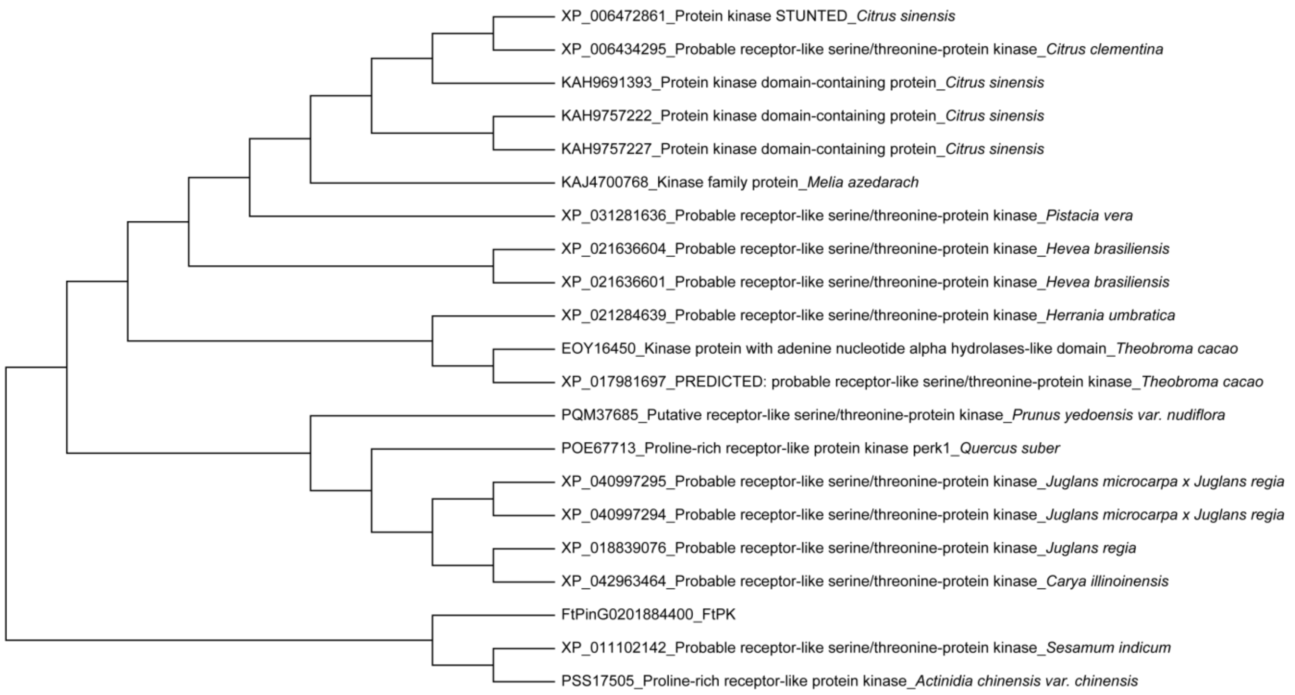

Figure S15. FtPK phylogenetic tree based on the neighbor-joining method tree using full-length amino acid sequences of orthologues genes in Tartary buckwheat (*FtPK*) and other plants.

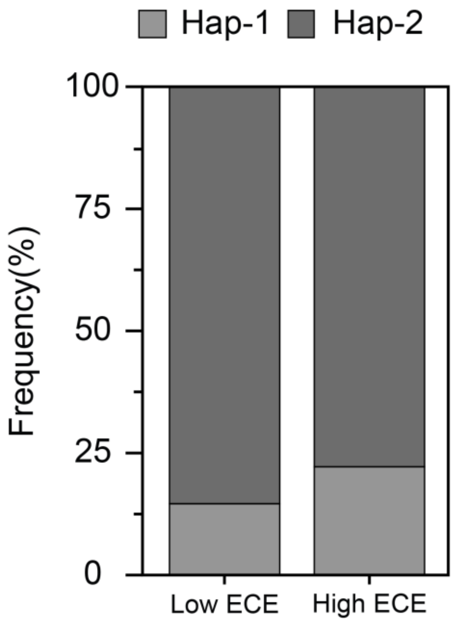

Figure S16. Frequencies of the two haplotypes in the low ECE and high ECE groups.(ECE: estimated Electrical Conductivity. The ECE has been used to indicate levels of sodium in soils).

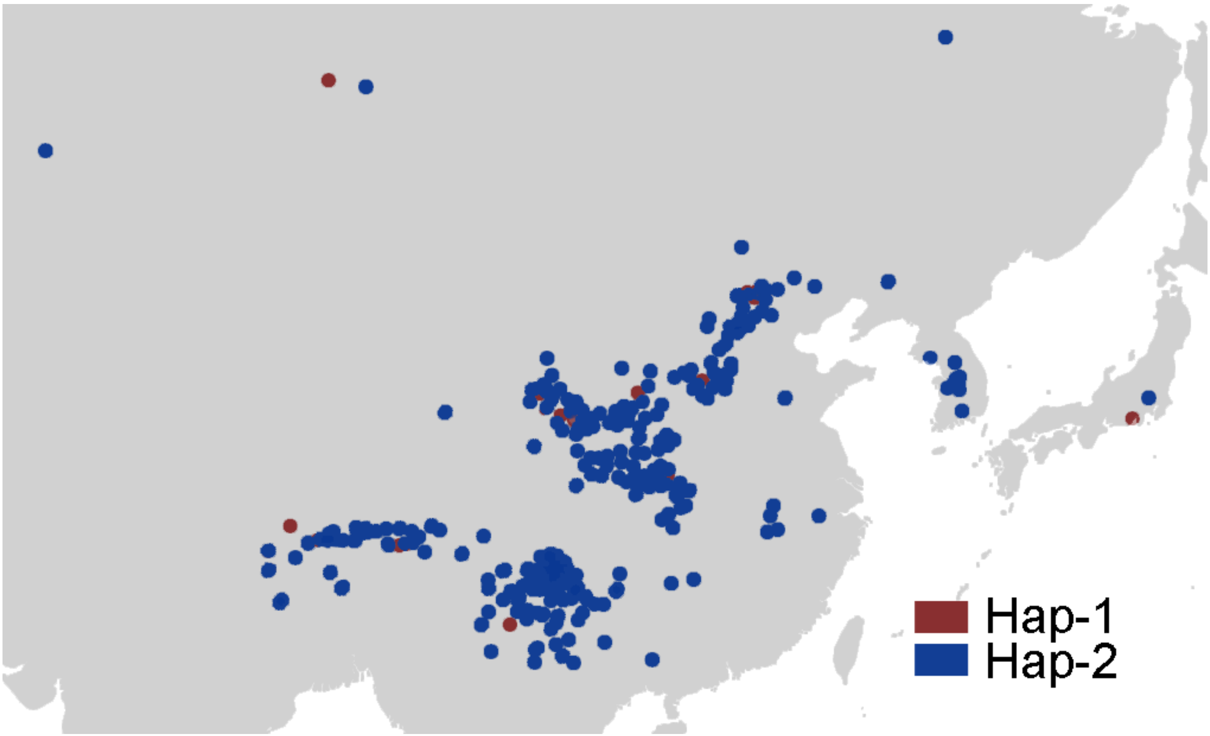

Figure S17. Geographic distribution of the Hap-1 (red) and Hap-2 (blue) Tartary buckwheat accessions.

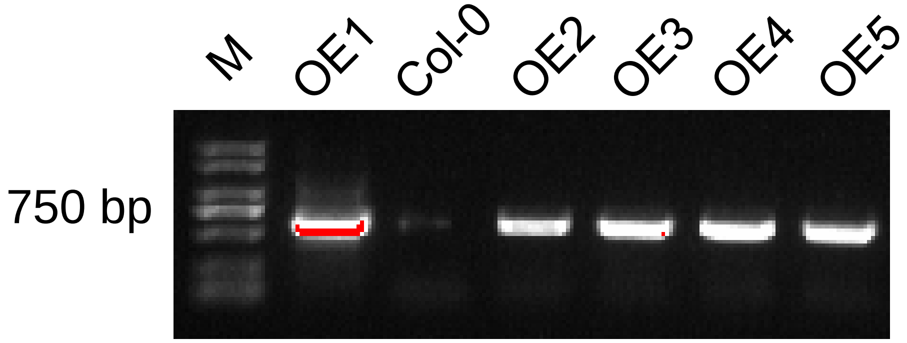

Figure S18. PCR analysis of *Arabidopsis* lines heterologously expressing *FtPK*.

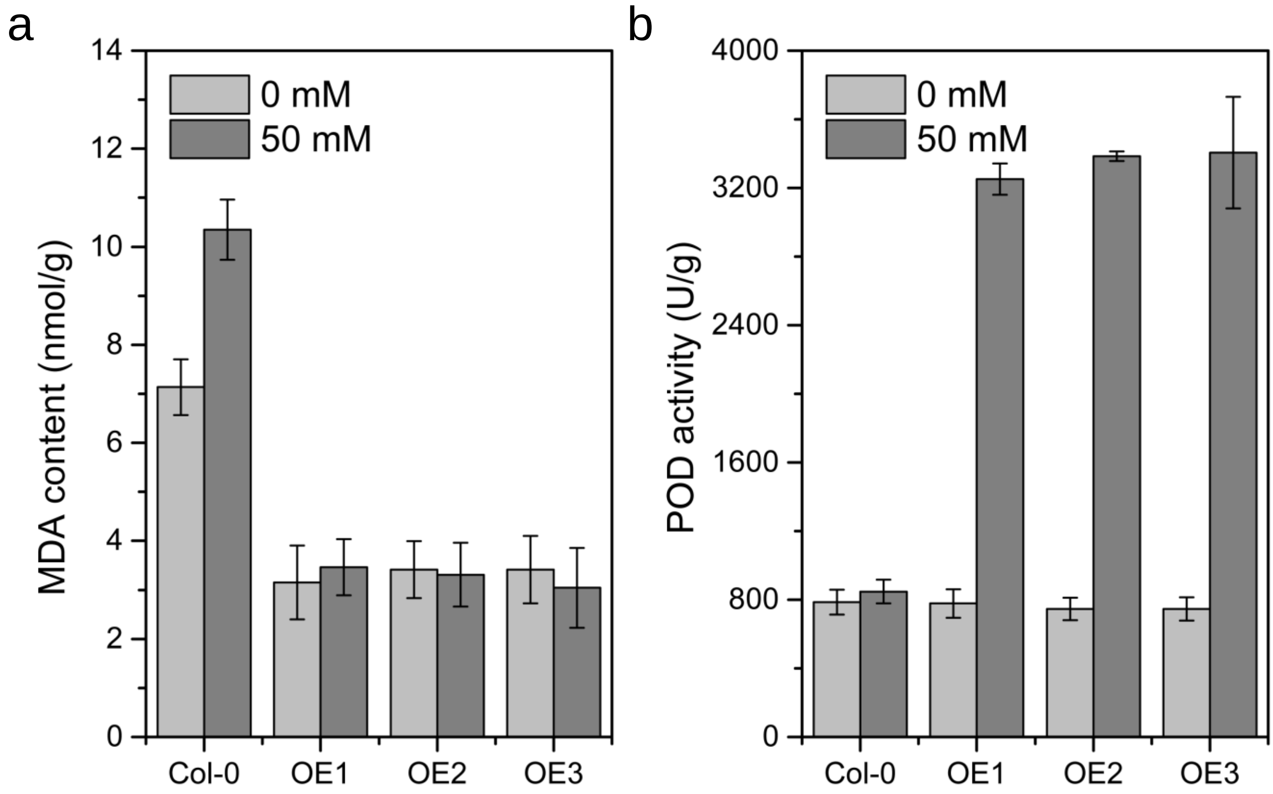

Figure S19. MDA content (a) and POD activity (b) in *Arabidopsis* heterologously expressing *FtPK* compared to WT with and without a salt treatment. Significant differences were tested using two-way ANOVA with Tukey HSD test. There was an effect of treatment (F = 7.988, df = 1, P = 0.012) and an effect of genotype (F = 101.627, df = 3, P = 0.000) on MDA content. And there was an effect of treatment (F = 1308.074, df = 1, P = 0.000) and an effect of genotype (F = 130.435, df = 3, P = 0.000) on POD activity.

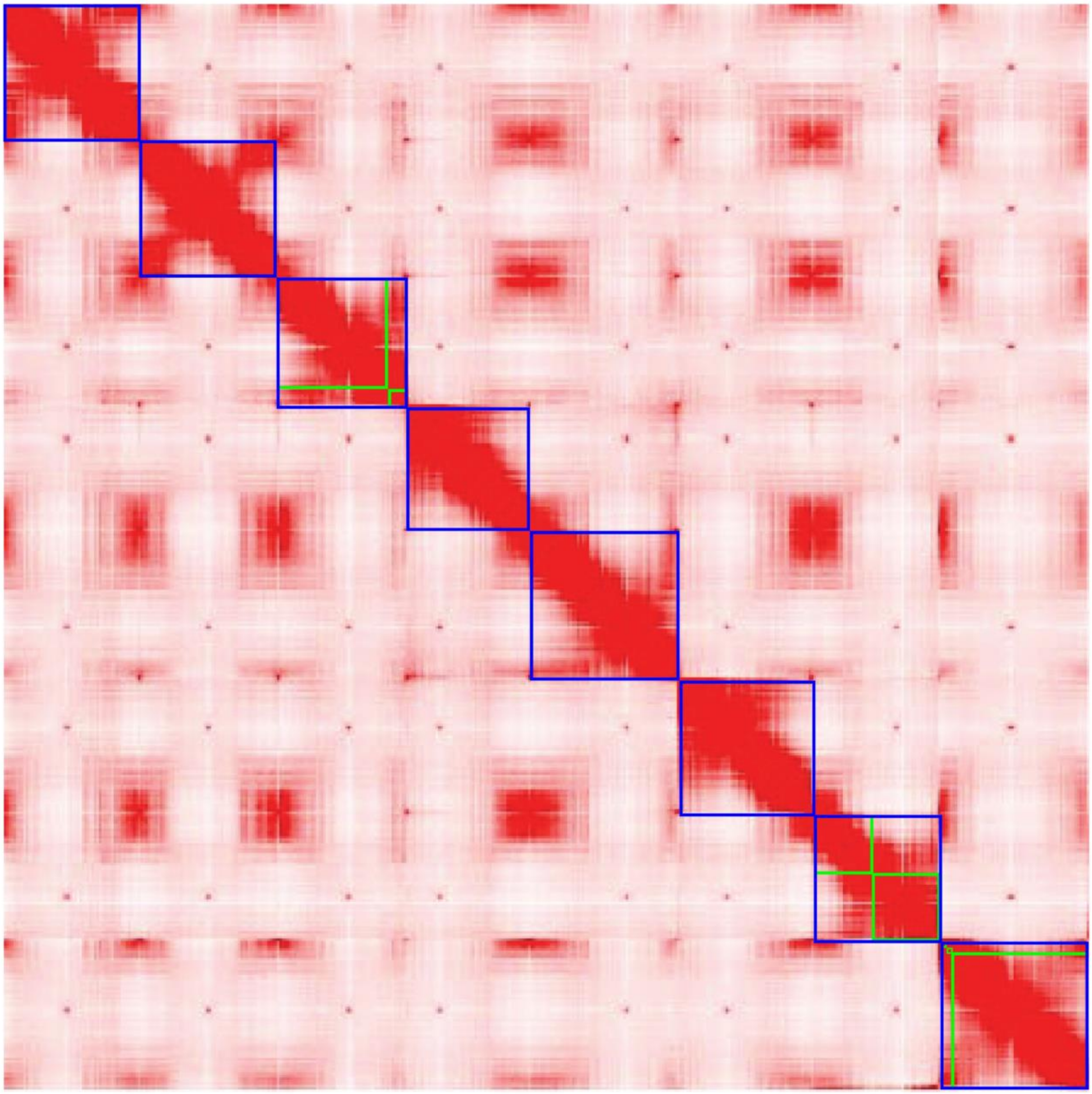

Figure S20. Hi-C contact matrix of the high-quality chromosome-scale genome assembly of EDT.

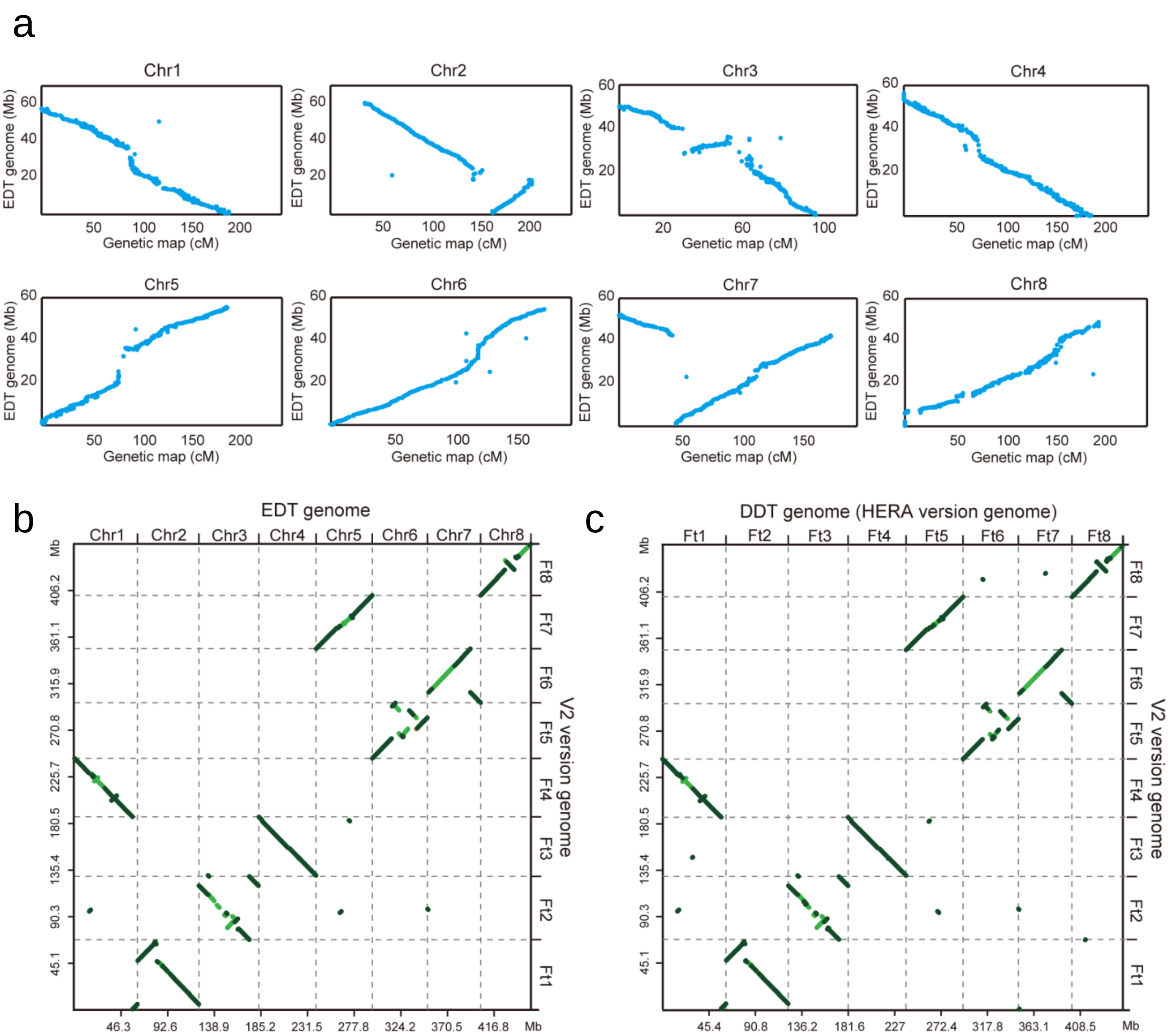

Figure S21. (a) Collinearity between the assembly and the genetic map of the RIL population. (b) Collinearity between the assembly of EDT and the V2 version assembly of Tartary buckwheat variety Pinku1 reference genome. (c) Collinearity between the assembly of HERA version assembly of Tartary buckwheat variety Pinku1 reference genome (DDT genome used in this study) and the V2 version assembly.

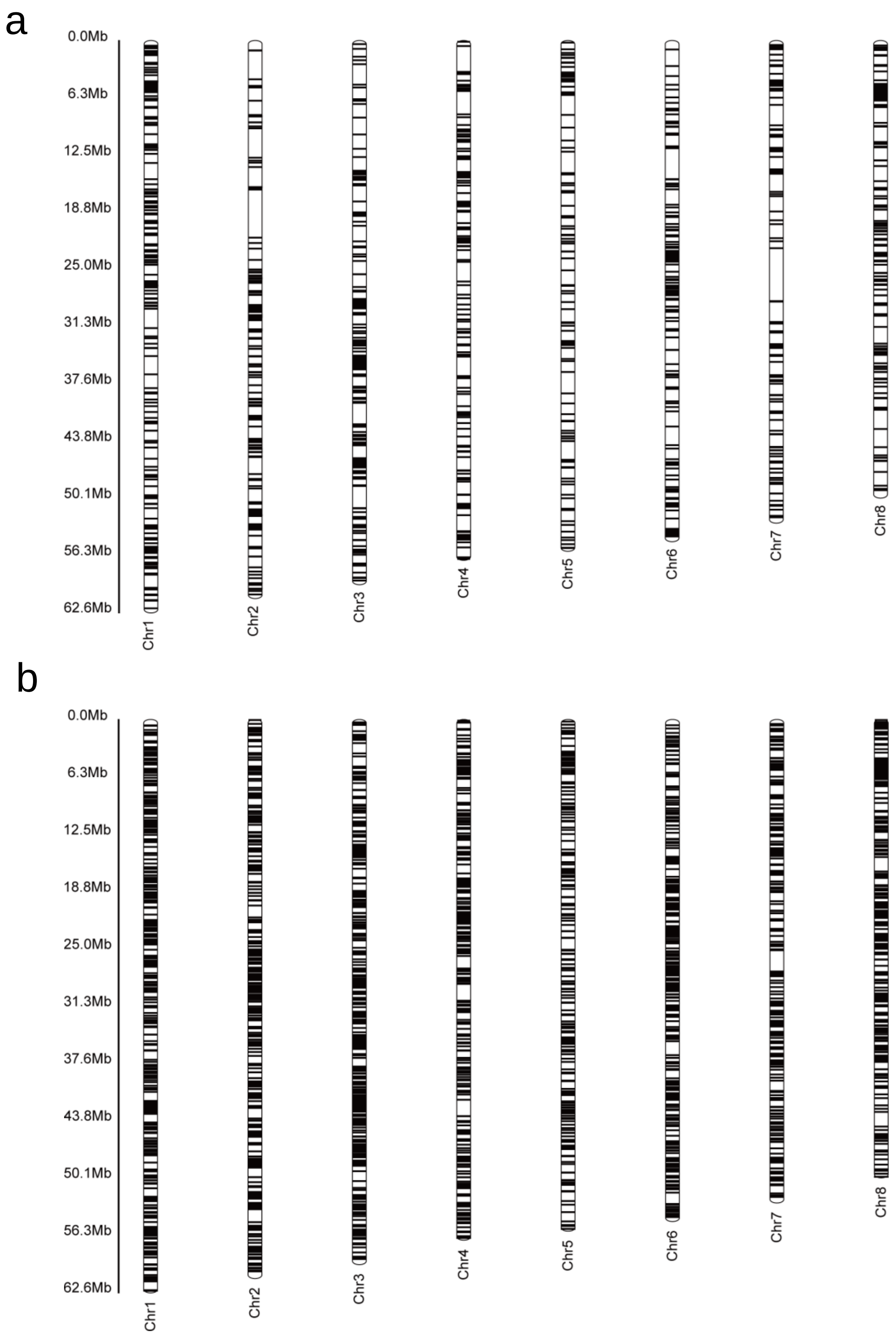

Figure S22. The distribution of deletions (a) and insertions (b) between EDT and DDT on eight chromosomes of Tartary buckwheat.
