# Supplementary material for: Genomic insight into the origin, domestication, dispersal, diversification and human selection of Tartary buckwheat
Source: Genome Biol. 2024 Feb 27;25:61. doi: 10.1186/s13059-024-03203-z (PMC10898187; doi:10.1186/s13059-024-03203-z)
Supplement: Supplementary file 3 — Additional file 3. Review history. [file 13059_2024_3203_MOESM3_ESM.docx]

**1^st^ round**

**Reviewer 1**

The manuscript of He et al. reports about population genomic analyses and genetic mapping in the pseudo-cereal buckwheat. The authors collected short-read resequencing data of a diversity panel, which they used for population structure analyses, selective sweep scans and GWAS. They analyzed several loci that were pinpointed by their genome scans in greater detail, mainly by genetic analysis in a heterologous system (A. thaliana). The latter fact somewhat limits the conclusions that can be drawn in buckwheat, but the authors' phrasing is generally circumspect. I found the paper an interesting read. It covers a range of topics related to the evolution of a crop that has not received as much attention as the major cereals. The authors provide a reasonable amount of evidence for their candidate genes and are careful not to claim too much. The display items are well-crafted. This is not a stellar piece of writing, but an expert in the field should be able to follow the authors' reasoning in most places. I have highlighted sections that need more explanations. The paper falls within the remit of Genome Biology and should be, if suitably revised, acceptable for publication in this journal.

1. I may have overlooked it, but I did not see a accession codes for the sequence data in INSDC archives. It would be good to add Biosample IDs to Table S1. Also the RIL data has to be submitted.
2. A Hi-C contact matrix should be shown to validate the structural integrity of the genome assembly. Collinearity between the assembly and a genetic map constructed de novo from the RIL sequencing data will provide additional confidence in the accuracy of the former.
3. I am not convinced about the Himalayan origin. What are the alternative hypotheses? How are these ruled out by the data shown in Fig. 1?
4. Line 248: The reasoning how Fig. 3c supports the Mongolian hypothesis should be made explicit. Quite a lot of profound things are supposed to be evident from this single figure panel, which is not an analysis result, but a schematic representation of the authors conclusions. As with the Himalayan origin: what are the competing hypotheses and how these ruled out?
5. The discussion is too long and lacks focus. It often only repeats what has been said already in the results or introduction sections. I was intrigued by the reference to wine-making in the abstract, but was then disappointed to find only a passing reference to it in the discussion.
6. line 87. The authors presuppose here that their readers know the difference between common and Tartary buckwheat.
7. lines 79-82 The two sentences basically say "Buckwheat is only grain crop in its genus. This is why it is rich in bioactive compounds." That doesn't make a lot of sense.
8. lines 119-110": I'm not a comparative linguist, but all the words in the Romance language refer to Saracens, important adversaries of the Crusaders. "Saracen" here might just refer to something that was introduced to Europe via an Islamic intermediary in the Middle East, rather than to Mongolian sarar.
9. line 130: "foreign" doesn't make a lot of sense in a journal with international readers, each of which has a different point of reference.
10. line 191: GRAS transcription factors are a whole family of genes.
11. line 193: The reasoning here is somewhat circular. The gene families alluded to in ll. 189-192 are involved in diverse processes, many of which can plausibly be linked to domestication. I'm convinced that this proves much about the reliability of the sweep scans.
12. Line 224: The conclusions read a bit odd. Disease resistance *reduction* is probably an unwanted side-effect of selection for unsavory metabolites after domestication. Also, the experiments in a heterologous do not tell us all that about the roles different alleles of the gene played during buckwheat evolution and whether such roles were "essential" as claimed by the authors.
13. Line 265: I can't follow the argument here. How does crop-wild gene flow *increase* the divergence between wild and domesticated populations?
14. Line 275: Flowering time regulation in which species?
15. Line 433: Domestication history cannot be subjected to "unequivocal demonstration". Our understanding of it can grow in accretive manner.

**Reviewer 2**

He et al. sequenced 78 new accessions of Tartary buckwheat and combined them with the previously published data (in total, 567 accessions from worldwide).
The study aimed to explore Tartary buckwheat's origin and disperse by performing population structure and gene flow. They also examine the selective sweep regions to investigate the genes involved in domestication and environmental adaption. Beyond that, they also collected HiC and PacBip data to de novo assemble a genome and did comparative genome analysis.
The topics addressed are interesting, but the manuscript has several problems as presented. Some of the conclusions are lack of evidence to support. Several issues with the manuscript need to be addressed before the manuscript is published.

Major issues:

1. The manuscript is disorganized, and the logic is not reasonable. For example, when talking about the origin of the Tartary buckwheat, then I would talk about the dispersal routes. So I would put the section "Dispersal of Tartary buckwheat followed routes of human migration " after the first section. Moreover, I would put Figure 3 as Figure 2. After discussing the origin and dispersion, you can discuss domestication and environmental adaptation. The "Human selection of easily dehulled Tatary buckwheat " section seems to be offset from the main story. Please think carefully and make it coherent with the storyline.

2. The authors concluded that a likely independent domestication process is driven by human intervention in the two genetically and geographically distinct groups (SL and NL). The conclusion is only based on very few overlapped selective sweep regions or genes identified by XP-CLR between SL and NL groups. This is not convincible because Mat et al., 2015 suggested that XP-CLR produces signals in the selected position but almost equally strong signals in other positions as well ( https://doi.org/10.1038/hdy.2015.42). If the author just based on XP-CLR, may add more false positive regions and get the wrong conclusion. I would use the de-correlated composite of multiple signals (DCMS) approach as Mat et al., 2015 developed to see how many overlapped selective regions between those two groups.

3. The author analyzed the selective sweep via two comparisons (HW-SL and HW-NL), the same as the Zhang et al., 2021 paper. I wonder how many regions that suffer from the selective sweep from this manuscript overlapped with the previous study? How many of them are different? Why? I did not see any mentions of either
Results or discussion. Again, is FtGULO detected in the previous study of Zhang et al. paper? How did the author calculate the disease index? What does the value indicate? Why is only 25% of the resistant type in the HW population? Is that because this gene is located in the introgression from the domesticated type?

4. What precisely the sample size? The number in the results is 567 accessions, but those mentioned in the methods and Table S1 disagree. Please clarify the sample size in the manuscript.

5. I will briefly introduce each subpopulation in the first section of the results. How did you name and classify them? Based on the GPS or what? In Figure 1a, I would use Haplotype distribution and frequency and haplotype network to show the geographic distribution of the demes and how they related to each other. Those figures will be much more precise to show the demes' origin and how they differentiated.

6. The authors used VCFtools to calculate Fst. VCFtools were designed to calculate the population genetic statistics for diploid samples. Still, the majority of the samples in this study are highly selfing (which will be treated as haploid), and can not VCFtools to calculate Fst.

7. All of the scripts and analytical tools used for a primarily computational project like the work reported here must be made available through Github, Bitbucket, or a similar version control repository. Because the scripts are essential to reproducing this work, they should be available to reviewers and readers. These resources are essential in part because samtools, GATK, and similar utilities include many options that alter the resulting output.

8. About the GWAS analysis, what is the rational of the criteria? If you set MAF >= 0.05 (567*0.05=28, still big enough), that will lose a lot of SNPs, which may be relative rare but may still have effect!

Minor issues:
1. Line 248, "based on centering the sampling coordinates using K-means clustering ". What did you base on for calculating the K-mean?
2. Line 194, what do you mean by "domestication sweeps "? As a population geneticist, I have never heard this term. Please explain.
3. Line 317, "To investigate the genetic basis of " what?
4. Line 346-349 shows 11 genes showing DE of the expression; why did the author focus on XIP, Mq02.g08037? How about the rest of the ten genes?
5. Line 485, "With the significance threshold set to P = 1×10−5. " is not a complete sentence.

**Reviewer 3**

This is a great study that looks into the domestication process of Tartary buckwheat. It has population genomic analysis to use evolutionary methods to infer the domestication history and combined this with quantitative genetics especially look at genetic variants associated with domestication traits. I think such combination of population and quantitative genetics approach is a very powerful way to understand the genomic basis of a crop domestication. And especially given the under researched status of Tartary buckwheat I think this paper is an important study that's very appropriate to publish in Genome Biology.

I have a lot of comments that are mainly for the authors to carefully explain or clarify a bit more what they say in the manuscript. I do have one major comment that I feel bad for bringing up given that the majority of this study I very enjoyed reading. I basically don't think their Fig4 results are showing any meaningful results to suggest they discovered a salt tolerance allele. Yes they find significant p-value, which they show is very marginally significant that I can't determine if their results are going to uphold actual genetic perturbation studies. Fig4 results in my opinion is the weakest point of this manuscript that I'm not even sure its necessary. Would like to see what the authors think and if they have any other data to definitively show that the region is a salt tolerance loci.

Else here are my comments.

Fig 1a, may be want to label what the different color points are.

Fig 2a, I think the authors need to clarify why there are two different colors for each Manhattan plot.

For Fig2a & b it's a nice result and I like the overlapping with domestication genes to show that those outliers of genetic differentiation with the wild genotypes are potential domestication loci regions. But it's a bit head scratching to show that results with the immunity results in Fig2c to n because the resistance result suggest to me that FtGULO isn't a domestication gene. Its potentially a gene that lost is wild variation and its making the crop succeptible to disease. And this doesn't sound like a domestication gene and it seems like it goes against what the authors set out as explained in L199-200. In fact FtGULO doesn't really look like an outlier region even in Fig2a & b. It seems marginal at best? Also the resistance allele isn't even fixed in the HW wild population so what is a genetic differentiation scan accomplishing here? All I'm saying here is that Fig2a & b could maybe be its own figure panel and the authors might think of highlighting actual domestication regions (as they've indicated in L189-195) they detected in their differentiation scan. Maybe show the zoom up of those regions since its hard to see what the XP-CLR statistics look like at the local gene level.

Fig 2j I don't think I understand the results of this figure correctly. The EV is displaying signals almost the same as a FtGULO-GFP fusion line. Doesn't that mean you can't make any conclusion? Why is an empty vector showing fluroscence as same as your line with a vector interest? I think some clarification in main text might help?

L232, sistant? What does that word mean? I don't think it's a word commonly used to describe phylogenetic and population relationships.

L232-234 I don't think I understand any of this sentence. What am I seeing in Fig3a that suggests a "sistant" (I still don't know what that means) relationship? And what is the qpGraph confirming? I don't think I understand anything being described here. And I was part of the study that conducted admixture graphs in rice to understand the dispersal of rice domestication. I would highly recommend the authors to re-examine this part of their manuscript to clarify what they mean.

How confident are the authors with their divergence time estimates? It's a very noisy estimate usually, and especially at these very recent time points in my experience they become really really noisy. Do the authors have any other validation or any other methods to show their times are robust? Some people (including us) have looked at past effective population size changes as another way to look at domestication history.

L248, what is this K-means cluster of? I think a clarification is needed.

Fig3c, there are arrows suggesting directionality in their inference. But how did they determine directionality? For instance, what is the evidence that the direction is from G5 to G6 and not from G6 to G5?

Grouping individuals into discrete populations are often messy and in times lead to false positive signatures of gene flow. For instance if you misgrouped an individual that was geographically (and genetically) borderline and grouped it as NLN when it should have been SL1 then you would see false signals of gene flow. Swarts et al (https://pubmed.ncbi.nlm.nih.gov/28774930/) have used Silouette scores and pruning those potentially misclassified individuals as way to make a "pure" population and test gene flow. I'd recommend the authors to also check for this as well.

For Fig4 results I am very surprised to see such small differences between Hap1 and Hap2 in the FtPK region for salt tolerance when the GWAS results show the FtPK has the most significant p-val across the chromosome. I could even argue p=0.0.24 in Fig4d is not significant after multiple hypothesis correction (which by the way is not clear how the authors have dealt with multiple hypothesis correction for all their result and not just the GWAS results). In fact Fig4 g,j and k all the results seem like the FtPK effect are marginal at best. The allele frequency between NL and SL isn't really that different it seems (fig4h) but despite this the authors see this region as an XP-CLR outlier (Fig4a)? Sure the gene expression is the only evidence that shows a big difference between Hap1 and Hap2 but I'm sure if you pick any random gene you could also see that level of difference. All this to say, I completely understand the work the authors must have done to get this result, but it just doesn't seem that convincing its that gene FtPK has any major effect on salt tolerance. Marginal at best, random noise maybe, false positive at worst case. Thoughts?

Fig5B you can barely see things on this plot. I'm not sure what I'm suppose to be seeing.

Fig 5f I think the authors need to better explain this figure. Why is there five rectangles of rows. And why does each rectangle have more squares? And why do some rectangles have a gene label and some don't? I don't think I can interpret what the figure is suppose to mean.

**Authors’ response**

Reviewer 1

***Comment 1.*** *I may have overlooked it, but I did not see a accession codes for the sequence data in INSDC archives. It would be good to add Biosample IDs to Table S1. Also the RIL data has to be submitted.*

**Response 1.** Thanks for your suggestion. We have added an "availability of data and materials" section illustrating the accession codes for the sequence data and RIL data (L795-L802). We have also added the Biosample IDs of resequencing accessions to Additional file 2: Table S1.

***Comment* 2.** *A Hi-C contact matrix should be shown to validate the structural integrity of the genome assembly. Collinearity between the assembly and a genetic map constructed de novo from the RIL sequencing data will provide additional confidence in the accuracy of the former.*

**Response 2.** Thanks for your suggestion. We have added the Hi-C contact matrix as Additional file 1: Fig. S20. The results exhibited the genome assembly possess a good structural integrity. We also added a figure showing collinearity between the assembly and the genetic map of the RIL population (Additional file 1: Fig. S21). Although some inversions and chromosome number difference, these are because the reference genome used for genetic map construction of the RIL population is the V2 version (Zhang et al., 2017), while the reference genome used for EDT genome assembly is the HERA version (Du et al., 2019). The collinearity analysis showed that not only the assembled genome of EDT but also the HERA version genome of Pinku1 exhibited corresponding inversions and chromosome number difference compared to the V2 version genome, confirming the collinearity between the assembled EDT genome and the genetic map of the RIL population.

***Comment*** ***3.*** *I am not convinced about the Himalayan origin. What are the alternative hypotheses? How are these ruled out by the data shown in Fig. 1?*

**Response 3.** Thanks for your suggestion. Initially, De Candolle speculated that Tartary buckwheat originated in northern China based on the existence of wild buckwheat by Russian taxonomists (De Candolle, 1883). However, he also pointed out that the existence of wild buckwheat in such area is suspect and no one has confirmed its existence. Subsequently, Japanese researcher Ohnishi found large amounts of wild buckwheat relatives were distributed in southern China, suggesting southern China might be the origin of buckwheat (Ohnishi, 1998). Using allozyme variability analysis, Ohnishi speculated the northwest part of Sichuan province is the origin of center of Tartary buckwheat (Ohnishi, 1998). Then, by utilizing molecular markers, Ohnishi illustrated the original birthplace of cultivated Tartary buckwheat is eastern Tibet and northwestern Yunnan province of China (Tsuji and Ohnishi, 2001). However, this research is based on the genetic variability obtained from 135 amplified fragment length polymorphism (AFLP) bands identified in 42 Tartary buckwheat accessions. In the present study, we use genome re-sequencing data identified 19,321,018 SNP from 567 Tartary buckwheat accessions. Both the sampling representativeness and the variations are greater than previous studies. Based on the population structure and the phylogenetic tree, wild relative species in *Fagopyrum spp*. as outgroup, we found accessions in HW group from the Himalayan region more likely to be the ancestor of cultivated Tartary buckwheat, and accessions in Yunnan and Sichuan province were mainly grouped in SL group, while accessions in northern China were mainly grouped in NL group. The HW group exhibited higher nucleotide diversity (π) and faster LD decay compared to SL and NL groups (Fig. 1d and 1e), suggesting the Himalayan region is more likely to be the origin center of cultivated Tartary buckwheat compared to northern China and Yunnan or Sichuan province in southern China. We have also revised the corresponding description in the introduction (L107-L116) and result section (L179-L188).

***Comment*** ***4.*** *Line 248: The reasoning how Fig. 3c supports the Mongolian hypothesis should be made explicit. Quite a lot of profound things are supposed to be evident from this single figure panel, which is not an analysis result, but a schematic representation of the authors conclusions. As with the Himalayan origin: what are the competing hypotheses and how these ruled out?*

**Response 4.** Thanks for your suggestion. Through population structure, maximum-likelihood phylogeny, PCA plots and population divergence shown in Fig. 1 and *f*_3_ statistics shown in Fig 2a, we found NLO exhibited closer relationship with NLI compared to HW and SL, suggesting Tartary buckwheat spread to Europe from northern China, rather than the Himalayas or southwestern China. Then, the *f*_3_ statistics revealed accessions collected from outside China (G8-G10) have closer genetic relationship with NL group (G5-G7) compared to HW (G1) and SL groups (G2-G4) (Fig. 2c), further confirm the above speculation. The phylogenetic tree (Fig. 2d) and pairwise fixation index (Fig. 2e) showed that individuals in NLO (G8-G10) have closer relationships with G6 (located in Inner Mongolia-Hebei) than other mini-groups in NL (G5 located in Qinghai-Gansu and G7 located in Hunan-Hubei-Jiangxi), supporting the hypothesis that Tartary buckwheat spread to Europe through the Mongolian region rather than other regions in northern China. Based on the divergence time (Fig. 2b; Additional file 1: Fig. S4) and effective population size (Additional file 1: Fig. S5) of five subgroups, we found NLI/NLO groups diverged at ca. 300-1,800 YBP, which was in accordance with the time of the westward expansion of the Mongol Empire. Thus, we speculated that Tartary buckwheat dispersed to Europe through the westward expansion of the Mongol Empire.

As for the Himalayan origin, results shown in Fig. 1 exhibited the HW group exhibited higher nucleotide diversity (π) and faster LD decay compared to SL and NL groups. In addition, the *f*_3_ statistics (Fig. 2c) showed the genetic relationship between HW and SL is comparable to that between HW and NL. Combining with the genetic diversity mentioned above, it is suggested that HW is the common ancestor of SL and NL. The phylogenetic tree (Fig. 2d) also showed that Himalayan region accessions (G1) possess a closer genetic relationship with outgroup compared to NLN (G5-G7) and NLF (G8-G10) accessions, further confirming that the Himalayan region is the center of origin of cultivated Tartary buckwheat.

***Comment*** ***5.*** *The discussion is too long and lacks focus. It often only repeats what has been said already in the results or introduction sections. I was intrigued by the reference to wine-making in the abstract, but was then disappointed to find only a passing reference to it in the discussion.*

**Response 5.** Thanks for your suggestion. The easily-dehulled type Tartary buckwheat was used for steaming as staple food, wine and tea making in areas settled by the Wa people. The easily-dehulled nature of EDT allows local Wa people to use ancient artificial wooden mortars and pestles to dehull Tartary buckwheat and steam together with rice as staple food to prevent lysine deficiency. We have revised the corresponding manuscript in the discussion section (L501-L505). In addition, in order to increase the significance of the discussion section, we also streamlined the discussion and added some customs related to Tartary buckwheat among the Yi people (L457-459).

***Comment*** ***6.*** *line 87. The authors presuppose here that their readers know the difference between common and Tartary buckwheat.*

**Response 6.** Thanks for your suggestion. Tartary buckwheat is self-pollinated, while common buckwheat is self-incompatible, making Tartary buckwheat more suitable for genetic diversity research than common buckwheat. In addition, due to the higher flavonoid content of Tartary buckwheat, it is generally considered that Tartary buckwheat has greater health protection efficacy than common buckwheat. Meanwhile, Tartary buckwheat has better high-altitude adaptability than common buckwheat, allowing it widely distributed in high-altitude mountainous areas. We have also added corresponding description in the introduction section (L87-L93).

***Comment*** ***7.*** *lines 79-82 The two sentences basically say "Buckwheat is only grain crop in its genus. This is why it is rich in bioactive compounds." That doesn't make a lot of sense.*

**Response 7.** Thanks for your suggestion. We have revised the corresponding description. For detail, please kindly see the introduction section of the revised version (L80-L85).

***Comment*** ***8.*** *lines 119-110": I'm not a comparative linguist, but all the words in the Romance language refer to Saracens, important adversaries of the Crusaders. "Saracen" here might just refer to something that was introduced to Europe via an Islamic intermediary in the Middle East, rather than to Mongolian sarar.*

**Response 8.** Thanks for your suggestion. Given the ambiguity in this name, we have deleted the description about Sarar.

***Comment*** ***9.*** *line 130: "foreign" doesn't make a lot of sense in a journal with international readers, each of which has a different point of reference.*

**Response 9.** Thanks for your suggestion. We have revised "foreign" as "outside China" (L140). In order to make international readers better understanding the manuscript, we also revised Northern Landrace-Native (NLN) and Northern Landrace-Foreign (NLF) as Northern Landrace-Within China (NLI) and Northern Landrace-Outside China (NLO), respectively.

***Comment*** ***10.*** *line 191: GRAS transcription factors are a whole family of genes.*

**Response 10.** Thanks for your suggestion. We have revised the manuscript as “some homologous of GRAS transcription factors” (L281).

***Comment*** ***11.*** *line 193: The reasoning here is somewhat circular. The gene families alluded to in ll. 189-192 are involved in diverse processes, many of which can plausibly be linked to domestication. I'm convinced that this proves much about the reliability of the sweep scans.*

**Response 11.** Thanks for your suggestion. We have revised the corresponding manuscript. For detail, please kindly see the results section of the revised version (L283-L284).

***Comment*** ***12.*** *Line 224: The conclusions read a bit odd. Disease resistance *reduction* is probably an unwanted side-effect of selection for unsavory metabolites after domestication. Also, the experiments in a heterologous do not tell us all that about the roles different alleles of the gene played during buckwheat evolution and whether such roles were "essential" as claimed by the authors.*

**Response 12.** Thanks for your suggestion. As the SNP was located in the promoter of *FtGULO* (Fig 3c), we speculated that different alleles may lead to the variation of the gene expression of *FtGULO*. Through qRT-PCR, we found the *FtFULO* expression in Tartary buckwheat accessions of Hap-A was significantly higher than that of Hap-T (Fig. 3f). Transient transcriptional activity assay further confirmed that the promoter of Hap-A has higher transcriptional activity than Hap-T. The disease resistance assay showed that *FtGULO* could improve disease resistance (Fig. 3m-n), suggesting this SNP could change the expression of *FtGULO*, thereby regulating plant disease resistance. In addition, we found this SNP was located in the selection sweep of HW vs SL and HW vs NL (Fig. 3a-b). And the frequency of Hap-A was significantly lower in SL and NL groups compared to HW group (Fig. 3i), we thus speculated that this SNP was involved in the disease resistance reduction during Tartary buckwheat domestication through regulating *FtGULO* expression. We have also revised the corresponding conclusion (L314-316).

***Comment 13.*** *Line 265: I can't follow the argument here. How does crop-wild gene flow *increase* the divergence between wild and domesticated populations?*

**Response 13.** Thanks for your suggestion. We have deleted the corresponding manuscript.

***Comment 14.*** *Line 275: Flowering time regulation in which species?*

**Response 14.** Thanks for your suggestion. Methyl-cpg-binding domain protein was responsible for flowering time regulation in Arabidopsis. We have also revised the corresponding manuscript (L326).

***Comment 15.*** *Line 433: Domestication history cannot be subjected to "unequivocal demonstration". Our understanding of it can grow in accretive manner.*

**Response 15.** Thanks for your suggestion. We have deleted "unequivocal". 

Reviewer 2

**Major issues:**

***Comment 1.*** *The manuscript is disorganized, and the logic is not reasonable. For example, when talking about the origin of the Tartary buckwheat, then I would talk about the dispersal routes. So I would put the section "Dispersal of Tartary buckwheat followed routes of human migration " after the first section. Moreover, I would put Figure 3 as Figure 2. After discussing the origin and dispersion, you can discuss domestication and environmental adaptation. The "Human selection of easily dehulled Tatary buckwheat " section seems to be offset from the main story. Please think carefully and make it coherent with the storyline.*

**Response 1.** Thanks for your suggestion. We have revised the section “Dispersal of Tartary buckwheat” as the second section and the section “Selection targets during domestication” as the third section. We also revised the order of the corresponding figures (Fig. 2 and 3). Easily dehulled Tartary buckwheat was a landrace grouped in SL1 group, which exhibited higher domestication degree compared to the other groups. However, there is only this one landrace with easily dehulled phenotype, population genetics could not reveal the genetic basis of this unique phenotype. So we assembled the genome of EDT and compared to the reference genome of a DDT variety. We have also added the corresponding description in the manuscript (L367-L374).

***Comment 2.*** *The authors concluded that a likely independent domestication process is driven by human intervention in the two genetically and geographically distinct groups (SL and NL). The conclusion is only based on very few overlapped selective sweep regions or genes identified by XP-CLR between SL and NL groups. This is not convincible because Mat et al., 2015 suggested that XP-CLR produces signals in the selected position but almost equally strong signals in other positions as well ( https://doi.org/10.1038/hdy.2015.42). If the author just based on XP-CLR, may add more false positive regions and get the wrong conclusion. I would use the de-correlated composite of multiple signals (DCMS) approach as Mat et al., 2015 developed to see how many overlapped selective regions between those two groups.*

**Response 2.** Thanks for your suggestion. We have added the result of DCMS analysis (Additional file 2: Table S9-S11). And we found 410 selective sweeps harboring 2,803 genes in HW-SL comparison, and 487 selective sweeps harboring 3,377 genes in HW-NL comparison. While only 153 selective sweeps harboring 785 genes were overlapped in both HW-SL and HW-NL comparison, further confirming the independent domestication process.

***Comment 3.*** *The author analyzed the selective sweep via two comparisons (HW-SL and HW-NL), the same as the Zhang et al., 2021 paper. I wonder how many regions that suffer from the selective sweep from this manuscript overlapped with the previous study? How many of them are different? Why? I did not see any mentions of either Results or discussion. Again, is FtGULO detected in the previous study of Zhang et al. paper? How did the author calculate the disease index? What does the value indicate? Why is only 25% of the resistant type in the HW population? Is that because this gene is located in the introgression from the domesticated type?*

**Response 3.** Thanks for your suggestion. Based on XP-CLR, we identified 462 genes located in 53 selective sweeps in HW-SL comparison and 416 genes located in 88 selective sweeps in HW-NL comparison overlapped with previous study (Additional file 2: Table S6, S7). The remaining 3,706 genes in HW-SL comparison and 3,319 genes in HW-NL comparison were newly identified in this study. DCMS analysis also found that most of the selective sweeps were not overlapped with previous study (Additional file 2: Table S9, S10). This was because we newly added 78 accessions, including 41 HW, 36 NL and 1 SL accessions, accounting for 57% (41/72) of HW, 12% (36/298) NL and 0.5% (1/197) SL accessions used in this study. The increasing number of HW accessions led to different selective sweeps identified compared to previous study. We have added the corresponding description in the result section (L267-L271).

*FtGULO* was located in the selective sweep in HW-SL comparison based on DCMS and π ratio in the previous study, implying the key role of this gene during Tartary buckwheat domestication. The disease index was calculated using the method described previously (Park et al., 2008; He et al., 2023). And the disease index is negatively correlated with plant disease resistance. We have also added the corresponding description in the method section (L652-653). Only 25% resistant haplotype were identified in HW group, which might be due to that it is a newly generated haplotype in HW group and has not yet introgression into the domesticated group. We have also added the corresponding description in the discussion section (L485-488).

***Comment 4.*** *What precisely the sample size? The number in the results is 567 accessions, but those mentioned in the methods and Table S1 disagree. Please clarify the sample size in the manuscript.*

**Response 4.** Thanks for your suggestion. We used 567 Tartary buckwheat accessions. In Additional file 2: Table S1, we also listed 7 species of outgroups, resulting in inconsistent sample sizes. We have revised the form of Additional file 2: Table S1 and unified the sample size in the entire manuscript.

***Comment 5.*** *I will briefly introduce each subpopulation in the first section of the results. How did you name and classify them? Based on the GPS or what? In Figure 1a, I would use Haplotype distribution and frequency and haplotype network to show the geographic distribution of the demes and how they related to each other. Those figures will be much more precise to show the demes' origin and how they differentiated.*

**Response 5.** Thanks for your suggestion. We have added the description about the name and classifications in the result section (L165-170) and revised Figure 1a accordingly.

***Comment 6.*** *The authors used VCFtools to calculate Fst. VCFtools were designed to calculate the population genetic statistics for diploid samples. Still, the majority of the samples in this study are highly selfing (which will be treated as haploid), and can not VCFtools to calculate Fst.*

**Response 6.** Thanks for your suggestion. Although VCFtools were designed to calculate the population genetic statistics for diploid samples, there are still references use this method to calculate F_ST_ for self-pollinated crops (Shang et al., 2022; Yang et al., 2022). Of course, we can also change the method if necessary.

***Comment 7.*** *All of the scripts and analytical tools used for a primarily computational project like the work reported here must be made available through Github, Bitbucket, or a similar version control repository. Because the scripts are essential to reproducing this work, they should be available to reviewers and readers. These resources are essential in part because samtools, GATK, and similar utilities include many options that alter the resulting output.*

**Response 7.** Thanks for your suggestion. We have deposited the scripts at the Github repository (https://github.com/Buckwheat-lab/Tartary_buckwheat_WGS). We have also added the corresponding description in the "Availability of data and materials" section (L802-803).

***Comment 8.*** *About the GWAS analysis, what is the rational of the criteria? If you set MAF >= 0.05 (567*0.05=28, still big enough), that will lose a lot of SNPs, which may be relative rare but may still have effect!*

**Response 8.** Thanks for your suggestion. We set MAF ≥ 0.05 based on the references (Cai et al., 2021; Li et al., 2021; Ma et al., 2021). We have also added the related references in the method section (L559).

**Minor issues:**

***Comment 1.*** *Line 248, "based on centering the sampling coordinates using K-means clustering ". What did you base on for calculating the K-mean?*

**Response 1.** Thanks for your suggestion. Geographic subsets of accessions were clustered using latitude and longitude coordinates by the K-means cluster method (Macqueen et al., 1967) with range extension less than 5 radius. We have also revised the corresponding description in the method section (L583-585).

***Comment 2.*** *Line 194, what do you mean by "domestication sweeps"? As a population geneticist, I have never heard this term. Please explain.*

**Response 2.** Thanks for your suggestion. We are sorry for these mistakes and have revised "domestication sweeps" as "selective sweeps ".

***Comment 3.*** *Line 317, "To investigate the genetic basis of " what?*

**Response 3.** Thanks for your suggestion. We have revised the corresponding manuscript as "To investigate the genetic basis of the easily dehulled phenotype of EDT" (L375).

***Comment 4.*** *Line 346-349 shows 11 genes showing DE of the expression; why did the author focus on XIP, Mq02.g08037? How about the rest of the ten genes?*

**Response 4.** Thanks for your suggestion. Although the other 10 genes are also differentially expressed, according to functional annotations, they are not directly related to plant cell wall biosynthesis (Additional file 2: Table S20). Xylan is the main component of cell walls, and xylanase inhibitors can directly regulate the formation of xylan. Therefore, we mainly focus on studying the function of FtXIP.

***Comment 5.*** *Line 485, "With the significance threshold set to P = 1×10−5. " is not a complete sentence.*

**Response 5.** Thanks for your suggestion. We have revised the corresponding manuscript as *"*The significance threshold was set at *P* = 1×10^−5^*"* (L561)*.*

Reviewer 3

***Comment 1.*** *I basically don't think their Fig4 results are showing any meaningful results to suggest they discovered a salt tolerance allele. Yes they find significant p-value, which they show is very marginally significant that I can't determine if their results are going to uphold actual genetic perturbation studies. Fig4 results in my opinion is the weakest point of this manuscript that I'm not even sure its necessary. Would like to see what the authors think and if they have any other data to definitively show that the region is a salt tolerance loci.*

**Response 1.** Thanks for your suggestion. Although the significant p-value of this locus is low, it is still the only locus that exceeds the threshold. In addition, we found that this gene is within the selective sweep between NL and SL not only through XP-CLR analysis (Additional file 2: Table S6, S7), but also DCMS analysis (Additional file 2: Table S9, S10), suggesting this gene is a key gene involved in NL and SL differentiation. We also analyzed the soil electrical conductivity in the location where these accessions obtained, and found this locus is related to the soil electrical conductivity (Additional file 1: Fig. S16). By combining the salt tolerance assay of overexpression materials (Fig. 4j and 4k) and the gene expression levels of *FtPK* in different haplotypes (Fig. 4e), we also confirmed that this gene is indeed involved in plant salt tolerance. Similarly, research was also conducted on the locus with marginally significant (Yu et al., 2023). Therefore, we think this locus remains to be studied and cannot be ignored due to its marginally significant.

***Comment 2.*** *Fig 1a, may be want to label what the different color points are.*

**Response 2.** Thanks for your suggestion. We have added the label of different color points.

***Comment 3.*** *Fig 2a, I think the authors need to clarify why there are two different colors for each Manhattan plot.*

**Response 3.** Thanks for your suggestion. We previously use different colors to distinguish the boundaries of different chromosomes. To make the figures easier to understand, we have unified the colors, and used short lines to represent the boundaries of different chromosomes.

***Comment 4.*** *For Fig2a & b it's a nice result and I like the overlapping with domestication genes to show that those outliers of genetic differentiation with the wild genotypes are potential domestication loci regions. But it's a bit head scratching to show that results with the immunity results in Fig2c to n because the resistance result suggest to me that FtGULO isn't a domestication gene. Its potentially a gene that lost is wild variation and its making the crop succeptible to disease. And this doesn't sound like a domestication gene and it seems like it goes against what the authors set out as explained in L199-200. In fact FtGULO doesn't really look like an outlier region even in Fig2a & b. It seems marginal at best? Also the resistance allele isn't even fixed in the HW wild population so what is a genetic differentiation scan accomplishing here? All I'm saying here is that Fig2a & b could maybe be its own figure panel and the authors might think of highlighting actual domestication regions (as they've indicated in L189-195) they detected in their differentiation scan. Maybe show the zoom up of those regions since its hard to see what the XP-CLR statistics look like at the local gene level.*

**Response 4.** Thanks for your suggestion. We have added local XP-CLR plots (Additional file 1: Fig. S10). And not only XP-CLR anlaysis (Additional file 2: Table S6, S7), but also DCMS analysis (Additional file 2: Table S9, S10) showed that *FtGULO* is located in the selective sweep. In addition, we also found this gene is also located in the selective sweep in previous published manuscript (Zhang et al., 2021; Additional file 2: Table S6, S7). These results suggested the important role of this gene during Tartary buckwheat domestication.

***Comment 5.*** *Fig 2j I don't think I understand the results of this figure correctly. The EV is displaying signals almost the same as a FtGULO-GFP fusion line. Doesn't that mean you can't make any conclusion? Why is an empty vector showing fluroscence as same as your line with a vector interest? I think some clarification in main text might help?*

**Response 5.** Thanks for your suggestion. EV exhibited the subcellular localization of GFP, which is located in cytosol and nucleus. If FtGULO-GFP is only localized in specific organelles (such as the nucleus), then fluorescence signals would only be observed in specific organelles (such as the nucleus). In Fig. 3j, FtGULO-GFP was located in both cytosol and nucleus. To make the figure easier to understand, we have revised EV to GFP.

***Comment 6.*** *L232, sistant? What does that word mean? I don't think it's a word commonly used to describe phylogenetic and population relationships.*

**Response 6.** Thanks for your suggestion. We have revised 'sistant' as 'distant' (L201).

***Comment 7.*** *L232-234 I don't think I understand any of this sentence. What am I seeing in Fig3a that suggests a "sistant" (I still don't know what that means) relationship? And what is the qpGraph confirming? I don't think I understand anything being described here. And I was part of the study that conducted admixture graphs in rice to understand the dispersal of rice domestication. I would highly recommend the authors to re-examine this part of their manuscript to clarify what they mean.*

**Response 7.** Thanks for your suggestion. For better understanding, we have carefully revised the corresponding manuscript. For detail, please kindly see the result section of the revised version (L197-L256).

***Comment 8.*** *How confident are the authors with their divergence time estimates? It's a very noisy estimate usually, and especially at these very recent time points in my experience they become really really noisy. Do the authors have any other validation or any other methods to show their times are robust? Some people (including us) have looked at past effective population size changes as another way to look at domestication history.*

**Response 8.** Thanks for your suggestion. We have added the past effective population size results (Additional file 1: Fig. S5), which was consistent with the estimated divergence time.

***Comment 9.*** *L248, what is this K-means cluster of? I think a clarification is needed.*

**Response 9.** Geographic subsets of accessions were clustered using latitude and longitude coordinates by the K-means cluster method (Macqueen et al., 1967) with range extension less than 5 radius. We have also revised the corresponding description in the method section (L583-585).

***Comment 10.*** *Fig3c, there are arrows suggesting directionality in their inference. But how did they determine directionality? For instance, what is the evidence that the direction is from G5 to G6 and not from G6 to G5?*

**Response 10.** Thanks for your suggestion. The phylogenic tree (Fig. 2d) was used to determine the relationship of accessions from northern China. It is showed that G5 is more closely related to G1 (Himalayan wild accessions) than G6. In addition, D-statistics (Additional file 2: Table S5) also showed a weak gene flow from G5 to G6, further confirming the direction is from G5 to G6.

***Comment 11.*** *Grouping individuals into discrete populations are often messy and in times lead to false positive signatures of gene flow. For instance if you misgrouped an individual that was geographically (and genetically) borderline and grouped it as NLN when it should have been SL1 then you would see false signals of gene flow. Swarts et al (https://pubmed.ncbi.nlm.nih.gov/28774930/) have used Silouette scores and pruning those potentially misclassified individuals as way to make a "pure" population and test gene flow. I'd recommend the authors to also check for this as well.*

**Response 11.** Thanks for your suggestion. We have added the Silhouette score results (Additional file 1: Fig. S6, S7), which showed that the groups can be well clustered.

***Comment 12.*** *For Fig4 results I am very surprised to see such small differences between Hap1 and Hap2 in the FtPK region for salt tolerance when the GWAS results show the FtPK has the most significant p-val across the chromosome. I could even argue p=0.0.24 in Fig4d is not significant after multiple hypothesis correction (which by the way is not clear how the authors have dealt with multiple hypothesis correction for all their result and not just the GWAS results). In fact Fig4 g,j and k all the results seem like the FtPK effect are marginal at best. The allele frequency between NL and SL isn't really that different it seems (fig4h) but despite this the authors see this region as an XP-CLR outlier (Fig4a)? Sure the gene expression is the only evidence that shows a big difference between Hap1 and Hap2 but I'm sure if you pick any random gene you could also see that level of difference. All this to say, I completely understand the work the authors must have done to get this result, but it just doesn't seem that convincing its that gene FtPK has any major effect on salt tolerance. Marginal at best, random noise maybe, false positive at worst case. Thoughts?*

**Response 12.** Thanks for your suggestion. We found that this gene is within the selective sweep between NL and SL, which was shown in XP-CLR analysis (Additional file 2: Table S12) and DCMS analysis (Additional file 2: Table S13), confirming this gene is essential in NL and SL differentiation. The small difference in allele frequency between NL and SL possibly due to the small sample size of Hap1. And we also found these SNPs were related to the soil electrical conductivity (Additional file 1: Fig. S16). If these SNPs do not have significant effects on salt resistance, we believe that there would not be such significant differences in expression levels and promoter activity between different haplotypes. Although the relatively high p-value (0.024) was obtained, it is still significant. And our experiments have also confirmed that this gene can improve plant salt tolerance, so we think this gene is still worth studying.

***Comment 13.*** *Fig5B you can barely see things on this plot. I'm not sure what I'm suppose to be seeing.*

**Response 13:** Thanks for your suggestion. We have revised this figure. For details, please kindly see Figure 5 of the new version.

***Comment 14.*** *Fig 5f I think the authors need to better explain this figure. Why is there five rectangles of rows. And why does each rectangle have more squares? And why do some rectangles have a gene label and some don't? I don't think I can interpret what the figure is suppose to mean.*

**Response 14.** Thanks for your suggestion. As we were unable to arrange 45 gene expression level in one row, we divided them into 5 rows. Each small square represents the differentially expression level of a gene between EDT and DDT. Square with label exhibited the differentially expressed genes. Square with label exhibited the differentially expressed genes. In order to better understand the figure, we have carefully revised the figure legends.

**References**

Cai X, Chang L, Zhang T, Chen H, Zhang L, Lin R, Liang J, Wu J, Freeling M, Wang X. Impacts of allopolyploidization and structural variation on intraspecific diversification in Brassica rapa. Genome Biol. 2021;31;22(1):166.

De Candolle A. Origine des Plantes Cultivées; G. Baillière et cie: Paris, France, 1883. Volume 43.

Du H, Liang C. Assembly of chromosome-scale contigs by efficiently resolving repetitive sequences with long reads. Nat Commun. 2019;10: 5360.

Li J, Yuan D, Wang P, Wang Q, Sun M, Liu Z, Si H, Xu Z, Ma Y, Zhang B, et al. Cotton pan-genome retrieves the lost sequences and genes during domestication and selection. Genome Biol. 2021;22(1):119.

Ma Z, Zhang Y, Wu L, Zhang G, Sun Z, Li Z, Jiang Y, Ke H, Chen B, Liu Z, et al. High-quality genome assembly and resequencing of modern cotton cultivars provide resources for crop improvement. Nat Genet. 2021;53(9):1385-1391.

Macqueen J. Some methods for classification and analysis of multivariate observations.Proc Symp Math Statist and Probability, 5th, 1967, 1.

Ohnishi O. Search for the wild ancestor of buckwheat III. The wild ancestor of cultivated common buckwheat, and of Tatary buckwheat. Economic Botany 1998;52:123–133.

Shang L, Li X, He H, Yuan Q, Song Y, Wei Z, Lin H, Hu M, Zhao F, Zhang C, et al. A super pan-genomic landscape of rice. Cell Res. 2022;32(10):878-896.

Tsuji K, and Ohnishi O. Phylogenetic relationships among wild and cultivated Tartary buckwheat (Fagopyrum tataricum Gaert.) populations revealed by AFLP analyses. Genes Genet Syst. 2001 ;76:47–52.

Yang T, Liu R, Luo Y, Hu S, Wang D, Wang C, Pandey MK, Ge S, Xu Q, Li N, et al. Improved pea reference genome and pan-genome highlight genomic features and evolutionary characteristics. Nat Genet. 2022;54(10):1553-1563.

Yu J, Zhu C, Xuan W, An H, Tian Y, Wang B, Chi W, Chen G, Ge Y, Li J, et al. Genome-wide association studies identify OsWRKY53 as a key regulator of salt tolerance in rice. Nat Commun. 2023;14(1):3550.

Zhang L, Li X, Ma B, Gao Q, Du H, Han Y, Li Y, Cao Y, Qi M, Zhu Y, et al. The Tartary buckwheat genome provides insights into rutin biosynthesis and abiotic stress tolerance. Mol Plant. 2017;10(9):1224-1237.

**2^nd^ round**

**Reviewer 1**

This is a tough call. The authors have revised their manuscript in many places and put together a detailed response letter.
Specifically, my techncial requests regarding the assembly have been addressed and a better explanation of the population genetic results has been given.
The other reviewers raised concerns about some experimental results. The authors tried hard to address these to keep the overall content of the revised manuscript largely unchanged compared to the original submission.
I believe a better course of action would have been to remove some of the contentious results or tone down the conclusions and state caveats clearly. This is a data-rich manuscript that even with a few subtraction would be valuable.
I have no specific suggestions for improvement and I am not opposed to acceptance if the other reviewers recommend it, too.

**Reviewer 2**

The author addressed majority of my comments; however, there are two comments are still not solved.

Comment 6. The authors used VCFtools to calculate Fst. VCFtools were designed to calculate the population genetic statistics for diploid samples. Still, the majority of the samples in this study are highly selfing (which will be treated as haploid), and can not VCFtools to calculate Fst.
Response 6. Thanks for your suggestion. Although VCFtools were designed to calculate the population genetic statistics for diploid samples, there are still references use this method to calculate FST for self-pollinated crops (Shang et al., 2022; Yang et al., 2022). Of course, we can also change the method if necessary.

Well, for sure, some references you listed still use vcftools to calculate the population statistics, but they did wrong! Not every publised paper get reviewed well by professional population geneticist! VCFtools were designed to calculate the population genetic statistics for diploid samples. Still, the majority of the samples in this study are highly selfing (which will be treated as haploid), and can not VCFtools to calculate Fst. You can either use angsd(10.1186/s12859-014-0356-4) or angsd-wrapper (0.1111/1755-0998.12578) to calculate that. They have haploid mode to calculate the Fst or pi.


Comment 8. About the GWAS analysis, what is the rational of the criteria? If you set MAF >= 0.05 (567*0.05=28, still big enough), that will lose a lot of SNPs, which may be relative rare but may still have effect!
Response 8. Thanks for your suggestion. We set MAF ≥ 0.05 based on the references (Cai et al., 2021; Li et al., 2021; Ma et al., 2021). We have also added the related references in the method section (L559).
Well, I know a lot of the publications used MAF ≥ 0.05 as threshold, but you need to decided based on your own sample size. Here you have576 individuals. If you set MAF >= 0.05 (567*0.05=28, still big enough), that will lose a lot of SNPs, which may be relative rare but may still have effect!

**Reviewer 3**

Thank you very much to the authors for responding to my comments. I have one last comment to make and if addressed I have no issues at all. Here is the interaction we had in previous review.

Comment 10. Fig3c, there are arrows suggesting directionality in their inference. But how did they determine directionality? For instance, what is the evidence that the direction is from G5 to G6 and not from G6 to G5?

Response 10. Thanks for your suggestion. The phylogenic tree (Fig. 2d) was used to determine the relationship of accessions from northern China. It is showed that G5 is more closely related to G1 (Himalayan wild accessions) than G6. In addition, D-statistics (Additional file 2: Table S5) also showed a weak gene flow from G5 to G6, further confirming the direction is from G5 to G6.

To me there is still nothing that suggests a direction of gene flow. The closeness of G5 and G6 is expected because they. have evidence of gene flow with each other. Coclustering on a phylogeny simply indicate phylogenetic closeness, nothing about direction of gene flow. Also they claim D-statistics shows a "weak gene flow from G5 to G6" but D-statistics do not tell you the direction of gene flow. I will retract my comment if the authors can show or find any study that showed a simple D-statistics show the directionaility of gene flow.

Look I think the authors could take a step back and report the data as is. If the authors want to interpret and synthesize there results in discussion section to say that "its likely that G5 gave genes to G6 because of these reasons and hypothesis", I have no problem with that. But the analysis they have done can not tell you the direction of gene flow. I;m always open to change my mind if they can show results

**Authors’ response**

Reviewer 1

***Comment 1.*** *This is a tough call. The authors have revised their manuscript in many places and put together a detailed response letter. Specifically, my techncial requests regarding the assembly have been addressed and a better explanation of the population genetic results has been given. The other reviewers raised concerns about some experimental results. The authors tried hard to address these to keep the overall content of the revised manuscript largely unchanged compared to the original submission. I believe a better course of action would have been to remove some of the contentious results or tone down the conclusions and state caveats clearly. This is a data-rich manuscript that even with a few subtraction would be valuable. I have no specific suggestions for improvement and I am not opposed to acceptance if the other reviewers recommend it, too.*

**Response 1.** Thanks for your suggestion. We have considered your suggestion and removed some of the contentious results during this revision.

Reviewer 2

***Comment 6.*** *Well, for sure, some references you listed still use vcftools to calculate the population statistics, but they did wrong! Not every publised paper get reviewed well by professional population geneticist! VCFtools were designed to calculate the population genetic statistics for diploid samples. Still, the majority of the samples in this study are highly selfing (which will be treated as haploid), and can not VCFtools to calculate Fst. You can either use angsd(10.1186/s12859-014-0356-4) or angsd-wrapper (0.1111/1755-0998.12578) to calculate that. They have haploid mode to calculate the Fst or pi.*

**Response 6.** Thanks for your suggestion. We attempted to calculate Fst using the two methods you mentioned. But both of these methods used raw data as imput, which is too time-consuming. We have been calculating for over a month, but still haven’t obtained the results. Thus, we attempted to use another set of Python scripts (https://github.com/simonhmartin/genomics_general/popgenWindows.py) to calculate Fst with the parameters set as -w 100000, -s 10000, -f haplo. The results showed that although Fst values changed (Figure 1d, Additional file 2: Table S3), it is still consistent with the trend of genetic distance between populations we previously calculated use VCFtools. Of course, if you still feel the method we used is not suitable, we are also continuing to use the methods you mentioned. But we need more time to get the final results.

***Comment 8.*** *Well, I know a lot of the publications used MAF ≥ 0.05 as threshold, but you need to decided based on your own sample size. Here you have576 individuals. If you set MAF >= 0.05 (567*0.05=28, still big enough), that will lose a lot of SNPs, which may be relative rare but may still have effect!*

**Response 8.** Thanks for your suggestion. We have set MAF >= 0.01 (For disease resistance, the number of accessions with phenotype is 320, 320*0.01 = 3.2, Fig. 3c, Additional file 2: Table S12; For salt stress, the number of accessions with phenotype is 151, 151*0.01 = 1.51, Fig. 4b, Additional file 2: Table S16). The results showed that the genes we focused on were still within the significant locus.

Reviewer 3

***Comment 10.*** *To me there is still nothing that suggests a direction of gene flow. The closeness of G5 and G6 is expected because they have evidence of gene flow with each other. Coclustering on a phylogeny simply indicate phylogenetic closeness, nothing about direction of gene flow. Also they claim D-statistics shows a "weak gene flow from G5 to G6" but D-statistics do not tell you the direction of gene flow. I will retract my comment if the authors can show or find any study that showed a simple D-statistics show the directionaility of gene flow.*

*Look I think the authors could take a step back and report the data as is. If the authors want to interpret and synthesize there results in discussion section to say that "its likely that G5 gave genes to G6 because of these reasons and hypothesis", I have no problem with that. But the analysis they have done can not tell you the direction of gene flow. I;m always open to change my mind if they can show results.*

**Response 10.** Thanks for your suggestion. Based on geographical distribution, G5 is closer to the origin center (where G1 located) than G6 and G7, and is more likely to be the ancestor of G6 and G7. Further, through phylogeny we found individuals in G5 were closer to their ancestors than other individuals in G6 and G7 (Fig. 2d). We thus used D-statistics analysis regarding G5 as the ancestor of G6 and G7 (Additional file 2: Table S5). And the results showed that D (G9, G6, G5, Outgroup) = 0.00188 ( > 0), suggesting a gene flow from G5 to G6, rather than from G6 to G5. However, due to the Z score < 3, we did not annotate gene flow in Additional file 2: Table S5.

Similarly, in D-statistics illustrated in Dong et al., 2023 (Figure 4B, Table S30), they regarded Syl-W1 as the ancestor, and found D (CG1, CG3/CG4/CG5, Syl-W1, Outgroup) > 0, suggesting gene flows from Syl-W1 to CG3/CG4/CG5. In addition, there are also some studies identify gene flow without considering the significant based on Z score (Kang et al., 2021; Figure 3d, Supplementary Table S26). However, to ensure the rigor of the results, as some gene flows were not significant (Z score < 3), we deleted some arrows in Fig. 2g. We also added some description in the discussion section “as phylogeny showed individuals distributed in Qinghai-Gansu province (G5) were closer to their ancestors than other individuals distributed in Inner Mongolia-Hebei province (G6) and Hunan-Hubei-Jiangxi province (G7), and D-statistics exhibited a week gene flow (Z < 3) from individuals distributed in Qinghai-Gansu province (G5) to that distributed in Inner Mongolia-Hebei province (G6), implying gene transfer between individuals in Qinghai-Gansu (G5) and Inner Mongolia-Hebei province (G6; Line 473-479).

**References**

Dong, Y., Duan, S., Xia, Q., et al. (2023). Dual domestications and origin of traits in grapevine evolution. Science ***379***, 892-901.

Kang, L., Qian, L., Zheng, M., et al. (2021). Genomic insights into the origin, domestication and diversification of *Brassica juncea*. Nat. Genet*.* ***53***, 1392-1402.

**3^rd^ round**

**Reviewer 2**

The author has solved all of the issues I addressed. No more comments!

**Reviewer 3**

The author has satisfactorily responded to my previous review
